# Use of cDNA Tiling Arrays for Identifying Protein Interactions Selected by *In Vitro* Display Technologies

**DOI:** 10.1371/journal.pone.0001646

**Published:** 2008-02-20

**Authors:** Kenichi Horisawa, Nobuhide Doi, Hiroshi Yanagawa

**Affiliations:** Department of Biosciences and Informatics, Faculty of Science and Technology, Keio University, Yokohama, Japan; Université de Montréal, Canada

## Abstract

*In vitro* display technologies such as mRNA display are powerful screening tools for protein interaction analysis, but the final cloning and sequencing processes represent a bottleneck, resulting in many false negatives. Here we describe an application of tiling array technology to identify specifically binding proteins selected with the *in vitro* virus (IVV) mRNA display technology. We constructed transcription-factor tiling (TFT) arrays containing ∼1,600 open reading frame sequences of known and predicted mouse transcription-regulatory factors (334,372 oligonucleotides, 50-mer in length) to analyze cDNA fragments from mRNA-display screening for Jun-associated proteins. The use of the TFT arrays greatly increased the coverage of known Jun-interactors to 28% (from 14% with the cloning and sequencing approach), without reducing the accuracy (∼75%). This method could detect even targets with extremely low expression levels (less than a single mRNA copy per cell in whole brain tissue). This highly sensitive and reliable method should be useful for high-throughput protein interaction analysis on a genome-wide scale.

## Introduction

Protein display technologies [1], such as phage display [2], ribosome display [3]–[5], DNA display [6] and mRNA display [7]–[9], are powerful tools for construction and *in vitro* selection of large libraries of genotype-phenotype conjugates. These libraries can be affinity-screened *via* the protein moiety (phenotype) followed by decoding of the nucleic acid moiety (genotype) to identify the selected proteins. These display technologies have been employed not only for directed evolution of novel proteins and antibodies [10]–[12], but also for the screening of protein-protein [13], [14], protein-drug [15], and protein-DNA [16] interactions from randomly fragmented cDNA libraries. Development of totally *in vitro* display techniques, such as ribosome display [3]–[5] and mRNA display [7]–[9], based on cell-free translation systems has extended the scope of previous techniques for protein interaction analysis using living cells, such as the yeast two-hybrid method [17] and biochemical methods coupled with mass spectrometry [18], because the variety of testable interaction conditions is greater, and the *in vitro* techniques are applicable to cytotoxic proteins.

However, the display technologies have a common bottleneck in the final step of identifying the specifically selected protein sequences. The decoding is usually achieved by cloning and DNA sequencing, but the following difficulties arise: 1) Only a limited number of clones can be analyzed, and thus positive candidates whose contents in the selected library are less than a threshold determined by the number of analyzed clones are lost as false negatives. 2) Positive sequences with low contents in a library can be enriched by iterative rounds of affinity-selection, but lower-affinity binders compete with higher-affinity binders and therefore drop out of the screening. 3) DNA fragments which are injurious to cloning hosts, *e.g.*, cytotoxic sequences, may be lost. 4) Cloning and sequencing of a huge number of copies of selected sequences is redundant, cost-ineffective, and time-consuming. Although novel high-throughput DNA sequencing methods that require no bacterial cloning process have recently been reported [19], these techniques are not yet widely available.

**Figure 1:**
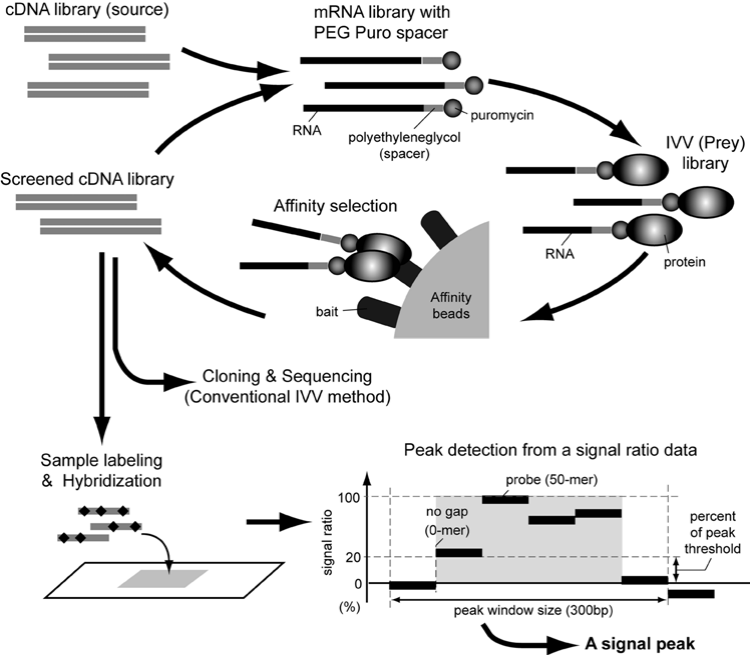
Scheme of iterative screening for protein interactions using the IVV method and a tiling array. A cDNA library was transcribed to an mRNA library, and then ligated with PEG Puro spacer [p(dCp)2-T(Fluor)p-PEGp-(dCp)2-puromycin] using T4 RNA ligase to prepare an IVV library. In this study, bait protein fused with tandem affinity purification (TAP) tag was co-translated with the IVV library in a wheat germ extract cell-free translation system. Complexes of bait protein and target IVV molecules were captured with affinity beads (IgG agarose) *via* the added TAP-tag. The RNA portion of the captured IVVs was reverse-transcribed and PCR-amplified. Finally, screened cDNA libraries were labeled and hybridized with tiling arrays as an alternative to cloning and sequencing (conventional IVV method).

To overcome the above limitations, we examined the use of a DNA microarray technique as an alternative to the cloning and sequencing processes (**Figure 1**). The combinatorial use of a tiling array [20] representing ORF sequences with *in vitro* display technology would provide a completely *in vitro* platform for highly sensitive and parallel analysis of protein interactions. It should be possible to detect enrichment of cDNA fragments of selected candidates even with low contents or low affinity. In this report, we demonstrate a highly sensitive analysis employing a transcription-factor tiling (TFT) array for identifying Jun-associated proteins selected with an mRNA display technology, *in vitro* virus (IVV) [21], [22], and show that the use of tiling arrays is indeed superior to the use of cloning and sequencing for decoding genetic information of proteins enriched by *in vitro* selection.

## Methods

### IVV screening

Preparation of bait template and IVV template libraries, and the procedure of IVV screening were described in detail in our previous report [21]. Details are also given in **Methods S1** online.

### Design and construction of the TFT array

Oligonucleotide arrays were constructed photolithographically by an oligo DNA microarray construction service (NimbleGen). The sequences of 1,562 mouse transcription regulatory factors listed by Gunji *et al.* [23], as well as 37 Jun-associated protein candidates found in our previous studies [14], [21], [22], were collected from the RefSeq (http://www.ncbi.nlm.nih.gov/RefSeq/) and Genbank (http://www.ncbi.nlm.nih.gov/entrez/query.fcgidbNucleotide) databases. Both strands of the total of 1,599 mRNA sequences were doubly represented by a total of 334,372 oligo DNA probes 50-mer in length, with no gap between the probes (**Figure 1**).

### Sample labeling, hybridization and signal detection

Biotin-labeling of the samples was performed by means of *in vitro* transcription from an SP6 promoter at the 5′-end of each cDNA fragment in the libraries, as described [24], with some modifications. In this process, biotin-labeled sense-strand RNA fragments were produced. Thus, only the antisense-strand probe set was further analyzed in this study. The labeled samples from the bait (+) and bait (−) screening were hybridized separately on the tiling arrays. The hybridized tiling arrays were stained with Cy3-Streptavidin (Amersham) and detection was done with a scanner. Details are given in **Methods S1** online.

### Data analysis

Collected data from the tiling array were normalized with the median correction algorithm. Ratios of signal values between the two samples from bait (+) and bait (−) screenings were calculated (**Data S1** online). The ratio data were expressed as log_2_X (X is the actual measurement). After signal measurement, specific signal peaks were identified by the “Windowed Threshold Detection” algorithm in SignalMap software (NimbleGen). This algorithm looks for at least four data points that are above a threshold value within a window. These points were grouped together and presented as a peak. We used the following parameters in the algorithm: Peak Window Size, 300 bp; Percent of Peak Threshold, 20% of maximum data in each mRNA sequence. The value of each peak was the maximum value of the data points in that peak. Only reproducible peaks in the duplicated data were collected as candidates for Jun interactors (**Table S1** online). A probe set for NM_183316 was not analyzed, because the sequence of NM_183316 overlapped with that of NM_025925 on the array.

### Real-time PCR analysis

Real-time PCR was performed with SYBR Premix Ex Taq (Takara) and protein-specific primer sets (**Table S2** online) on the LightCycler (Roche) as previously described [22]. The standard template DNA for the quantitative analysis was PCR-amplified from each selected sequence.

### *In vitro* pull-down assay

Pull-down assay using the C-terminal fluorescence labeling technique was performed as previously described [21], with some modifications. The bait Jun and prey proteins were translated *in vitro* separately. Only the preys were fluorescence-labeled in *in vitro* translation. The bait and preys were incubated with affinity beads, and captured prey proteins were electrophoresed and analyzed by using a Molecular Imager FX (Bio-Rad). Details are given in **Methods S1** online.

### Surface plasmon resonance analysis

SPR analysis was performed on a BIACORE3000 instrument with a CM4 sensor chip conjugating anti-GST mAb (Biacore). GST-fused candidate proteins were employed as ligands. Full-length mouse Jun fused with a His-tag was employed as an analyte. The experiments were performed under two conditions of analyte concentration (500 nM and 250 nM). Details are given in **Methods S1** online.

## Results

### Design of a transcription-factor tiling (TFT) array and sample labeling

In affinity selection of protein interactions from randomly fragmented cDNA libraries, relatively short cDNA fragments encoding specific binding regions are often obtained. In order to detect these fragments, we adopted a tiling array strategy for the design of custom oligo DNA microarrays as follows: 1) Oligonucleotide probes of 50-mer in length were used. This is the preferred length for microarray probes, because shorter probes result in low sensitivity and longer probes produce non-specific signals [25]. 2) There should be no gaps between the probes. A contiguous linear series of data is required to recognize a signal peak in the algorithm for tiling array analysis (in this case, at least 4 data points are needed in a search window), so the probes must be densely arranged. 3) mRNA sequences were employed for the tiling array. Only coding regions are required for the purpose of protein-interaction analysis, so other genomic sequences, *e.g.*, introns, control regions and non-coding RNAs, were not employed. In this study, we constructed TFT arrays containing ∼1,600 ORF sequences of known and predicted mouse transcription-regulatory factors (334,372 oligonucleotides) to analyze cDNA fragments from IVV screening for Jun (a transcription factor)-interactors [21].

We also improved the method for labeling of cDNA samples. Usually, double-stranded DNA samples for a tiling array analysis are labeled by using random primers [26]. However, cDNA fragments selected from a randomly fragmented cDNA library [21] seem to be too short for efficient labeling by random priming. Indeed, in a test analysis with a TFT array using the random priming labeling method, we failed to detect all of the previously detected candidates (data not shown). Therefore we employed another labeling procedure [24], in which sense-strand labeled RNAs were produced by one-step *in vitro* transcription using a SP6 promoter attached to cDNA fragments from IVV screening.

### Identification of selected candidates using the TFT array

**Figure 2:**
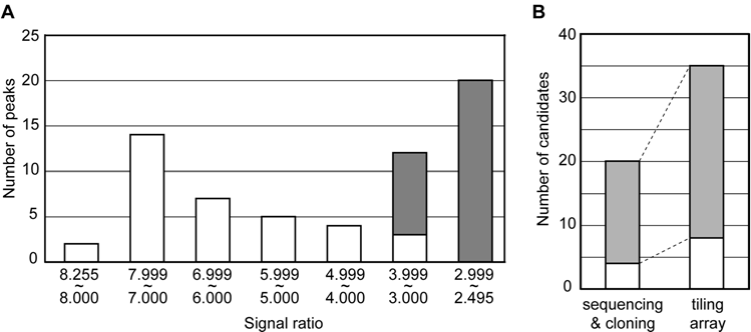
Data from the tiling array. (A) The top 10% candidates were confirmed by real-time PCR. White and gray indicate numbers of enriched and non-enriched candidates, respectively. (B) Numbers of known (white) and newly selected (gray) proteins from conventional sequencing and the TFT arrays.

From the 5th-round DNA library of the IVV screening in the presence and absence of a bait Jun protein, called bait (+) and bait (−) screening, respectively [21], we obtained labeled RNAs and hybridized them onto the tiling array. First, the ratios of the signal intensities from the experiments in the presence and absence of bait were calculated. The ratio data are presented in **Data S1** online as a GFF formatted file. Next, we searched for signal peaks in the data, as described in Methods. Only reproducible signal peaks were collected (**Table S1** online); the total number of peaks was 647 on 545 mRNA sequences (some of the mRNA sequences included multiple peaks). To distinguish between true positives and false positives, specific enrichment of the selected candidates was validated by real-time PCR. Among the top 10 percent of the peaks (64 regions), specific enrichment of 35 peaks was confirmed in the screening (white graph in **Figure 2A**; the signal intensity and peak data of the 35 candidates are presented in **Figure S1** online). The data indicate that the appropriate threshold for distinguishing between true positives and noise in the microarray signal is a signal ratio of 3∼4.

The 35 candidates identified in the present study include all of the 20 Jun-interactors identified in our previous studies using conventional cloning and sequencing (**Table 1**) [21], [22]. Furthermore, the 35 candidates include eight well-known Jun-associated proteins, *i.e.*, c-Maf, Fos, Jun, Atf7, Atf4, Jdp2, Atf3 and Fosl2 (**Table 1**), which is double the number in the previous study, in which four known Jun-interactors were obtained (white graphs of **Figure 2B**) [21], [22]. In other words, 15 proteins including four known Jun-interactors were newly detected using the TFT arrays.

**Table 1:** Thirty-five selected proteins

Gene symbol	Cloning & Sequencing	Accession no.	Signal ratio	Locus on mRNA sequence (base)	Abundance ratio in the initial library (%)	Abundance ratio in the 5th round library (%)	Enrichment
Nrbf2	+	NM_025307.2	8.255	651…950	5.7E-4	4.8E-1	840
4732436F15Rik	+	XM_143418.3	8.134	2051…2300	1.1E-4	1.5E+0	14,000
c-Maf*	-	S74567.1	7.796	1805…2004	6.4E-5	5.2E-1	8,000
SNAP19	+	NM_025925.1	7.734	1…250	1.6E-3	2.2E+1	14,000
Fos*	+	NM_010234.2	7.720	501…800	7.4E-5	1.5E+0	20,000
Mapre3	+	NM_133350.1	7.714	601…950	9.2E-4	1.4E+0	1,600
Cspg6	+	NM_007790.2	7.577	2151…2700	4.1E-4	1.0E+0	2,500
Kif5A (region C)	+	NM_008447.2	7.491	2251…2900	3.3E-3	4.3E+0	1,300
9130229H14Rik	+	XM_135706.4	7.487	51…400	1.6E-3	1.2E+0	730
Jun*	+	NM_010591.1	7.411	1701…1950	1.4E-4	4.3E-1	3,100
Mapk8ip3	+	NM_013931.1	7.282	1351…1800	7.3E-4	5.3E-1	720
Creb3	-	XM_131375.2	7.172	601…800	3.3E-4	2.6E-3	8
Kif5B (region C)	-	NM_008448.2	7.141	2801…3200	1.3E-3	1.2E-1	95
Nef3	+	NM_008691.1	7.129	951…1300	8.2E-3	1.5E+0	190
Kif5C (region C)	+	NM_008449.2	7.106	2701…3200	2.2E-3	6.4E+0	2,900
Eef1d	+	NM_029663.1	7.083	1301…1750	5.5E-3	2.3E+0	420
Atf7*	-	NM_146065.1	6.999	1051…1300	2.7E-5	1.4E-1	5,000
Atf4*	+	NM_009716.1	6.991	1001…1300	5.4E-4	1.9E+0	3,500
Cutl1	-	NM_009986.2	6.850	301…500	1.4E-3	3.2E-1	230
Jdp2*	+	NM_030887.2	6.768	451…700	7.1E-4	2.1E+0	3,000
Ofd1	-	NM_177429.2	6.692	1752…2001	1.4E-4	2.6E-1	1,800
GFAP	+	NM_010277.1	6.551	901…1100	1.1E-3	8.8E-2	77
Kif5C (region N)	+	NM_008449.2	6.098	1201…1450	1.2E-2	5.2E+0	450
Psmc5	-	NM_008950.1	5.961	51…300	3.5E-3	6.4E-2	19
Kif5B (region N)	+	NM_008448.2	5.937	1301…1550	6.4E-4	1.5E-1	230
Atf3*	-	NM_007498.2	5.574	401…950	1.1E-7	2.0E-2	180,000
B130050I23Rik	+	NM_153536.2	5.213	1151…1450	1.9E-4	1.5E-2	80
Cebpg	-	XM_133383.2	5.122	401…750	3.5E-5	2.7E-3	78
1200008A14Rik	+	NM_028915.1	4.623	1501…1750	3.6E-4	2.1E-1	600
Myh11	-	NM_013607.1	4.343	3251…3650	1.4E-4	2.9E-4	2
Tax1bp1	-	NM_025816.1	4.176	501…750	2.7E-3	1.0E-2	4
Myt1	-	NM_008665.2	4.084	3251…3550	1.1E-5	1.3E-4	12
Fosl2*	-	NM_008037.3	3.935	401…750	2.1E-5	1.4E-2	670
Tef	-	NM_017376.2	3.467	851…1000	1.3E-4	1.1E-3	8
Cebpz	-	NM_009882.1	3.012	1451…1750	1.2E-4	3.7E-4	3

* Previously reported interactors with Jun.

### Verification of the newly found protein-protein interactions

To confirm the physical association of the 11 newly discovered candidates with Jun, we first performed *in vitro* pull-down assay. Seven of the 11 tested candidates exhibited specific interaction with the bZIP domain of Jun (**Figure S2** online). The affinity of the remaining four proteins, *i.e.*, Cutl1, Myh11, Tax1bp1 and Cebpz, for Jun may be weaker, because their enrichment ratios (excluding that of Cutl1) in the IVV screening were lower than those of others (**Table 1**). Thus, we next employed the surface plasmon resonance (SPR) method, a highly sensitive analysis tool for protein interactions. In this case, all of the candidates except Tax1bp1 interacted with Jun in a concentration-dependent fashion (**Figure S3** online). Although most of the above tested interactions seem to be very weak, we considered that the interactions are true positives, because all of the candidates except Cebpz contain leucine-heptad repeats in the selected regions, and such repeats are an important motif for heterodimerization with Jun. Further experiments *in vivo* will be required to examine the physiological roles of these interactions.

## Discussion

To evaluate the quality of the interaction data, coverage and accuracy were calculated as follows. Jun interacts with many bZIP superfamily proteins and structurally unrelated transcription factors in a binary fashion. Chinenov and Kerppola comprehensively collected reported Jun-interactors in their review, and the number of mammalian Jun-interactors was 51 at that time [27]. Of the 51 interactors, some lack the potential to bind with the bait Jun protein in our experiment for various reasons. For example, the SMAD interacting region of Jun [28] was deleted from our bait protein construct, and NFAT family proteins require a DNA fragment including the AP-1 sequence [TGA(C/G)TCA] and NFAT recognition element (GGAAAA) for stable interaction with Jun [29]. Also, the expression of some of the interactors, *e.g.*, JunD, Fra1, Batf, MafA, Nrl and NF-IL16, was not confirmed in the cDNA library used in this study (data not shown). In all, 29 Jun-interactors were expressed in the cDNA library and were expected to bind with the bait Jun protein used here. Of these 29 proteins, four (14%) and eight (28%) were detected by the conventional sequencing and by the newly introduced TFT array method, respectively. This is a remarkable increase and confirms the value of our new methodology as a screening tool for protein interactions. While the coverage was increased considerably, the accuracy did not decrease. Specifically, the number of false positives did not increase: the rates of confirmation of proteins by *in vitro* pull-down assays in the previous and present studies were 75% and 74%, respectively. Further, we confirmed by SPR that most of the unbound candidates in the pull-down assay actually interacted with Jun. These results indicate that generation of false positives in this novel method is low, and that the method is practical. Undetected remaining interactors were considered to be false negatives. Mismatching of the selection conditions, *e.g.*, salts, detergents, and pH, or the bait construct, *e.g.*, length, region, and tags, might inhibit these interactions.

For quantitative analysis, the abundance ratios of 35 specifically selected candidates in the initial and screened cDNA libraries were determined by real-time PCR, and the enrichment rates (abundance ratio in the 5th round library per that in the initial library) were also calculated (**Table 1**). The abundance of the 15 newly found candidates (excluding c-Maf, Cutl1 and Ofd1) was less than the theoretical threshold determined from the results of our previous study (an analysis of 451 clones) [21], [22]. In order to detect the least abundant candidate (Myt1; 1.3×10^−4^% of the screened cDNA library) by cloning and sequencing, it would have been necessary to analyze at least 1.0×10^6^ clones. These results indicate that our new method is more sensitive, higher-throughput and more cost-effective than the previous method.

From the standpoint of the detection sensitivity, the combinatorial use of the IVV method with TFT arrays provides an extremely sensitive method for protein-interaction analysis, because even a very weakly expressed target, Atf3, could be detected in this study. In the cDNA library before IVV screening, the content of fragments of the selected region of Atf3 was 1.2×10^−7^%. If one mRNA molecule existed per cell, the content of a fragment of the gene would be about 1.2×10^−5^ to 5.9×10^−5^% (we employed the parameters from a reference for this calculation [30], and the details are given in **Methods S1** online). Thus, the content of Atf3 mRNA in the initial library corresponds to about one molecule per 20 to 100 cells. This suggests that Atf3 is expressed at a very low level in a cell type that is a minor component of whole mouse brain tissue. It is noteworthy that targets expressed at such low levels can be detected without the need for a cell purification procedure, *e.g.*, collection of somatic stem cells by flow cytometry. The high sensitivity of our method may allow access to targets which would be hard to analyze with other existing tools, *e.g.*, the TAP method [18].

Among the newly detected Jun-associated protein candidates, Cebpg, Creb3, and Tef are intriguing proteins as Jun-associated transcriptional regulators, because they contain basic regions near the leucine heptad motifs, which are necessary for binding with regulatory sequences on the genomic DNA; many known Jun-associated proteins contain such structures. Cebeg is a member of the CAAT/enhancer-binding protein (C/EBP) family, which is one of the largest and most highly conserved groups of eukaryotic transcription factors. Cebpg is known to interact with Cebpb, a member of the C/EBP family, but the function of the protein is not well understood [31]. Davydov *et al.* indicated that Cebpg binds to the positive regulatory element-I (PRE-I) of the human interleukin-4 gene by forming a heterodimer with Fos protein. However, the interaction between Cebpg and Jun was not clearly delineated [32]. Creb3 is also a transcriptional regulator belonging to the cyclic AMP response element-binding (CREB)/activating transcription factor (ATF) protein family. The bZIP region of Creb3 is strikingly similar to that of ATFa, a known partner of Jun [33]. Tef is a member of the proline and acidic amino acid-rich basic leucine zipper (PAR bZIP) transcription factor family. The PAR bZIP proteins control circadian rhythms in tissues such as the suprachiasmatic nucleus and the liver. Mice deficient in all three PAR bZIP proteins are highly susceptible to generalized spontaneous and audiogenic epilepsies that are frequently lethal [34]. No information is available about the functional relationship between Tef and Jun. More detailed studies *in vivo* may reveal novel and unexpected functions of Jun in combination with these proteins.

In summary, we have applied tiling array technology, which has previously been used for ChIP-chip assays [26] and transcriptome analyses [20], to protein-interaction analysis with an *in vitro* display technique for the first time. Compared with previous results obtained with cloning and sequencing [21], [22], use of the tiling array greatly increased the coverage of known Jun-interactors from 14% to 28% without any decrease of accuracy (∼75%). The new method can also detect targets expressed at extremely low levels. We believe that this highly sensitive and reliable method has the potential to be used widely, because the tiling array method can easily be extended to genome-wide scale, even though the search space is limited in tiled sequences, and the method can also be used in combination with other display technologies, such as phage display and ribosome display.

## Supporting Information

Data S1 All ratio data from the tiling arrays
(2.36 MB ZIP)

Figure S1 Signal ratio and peak data of selected candidates
(2.14 MB PDF)

Figure S2 *In vitro* pull-down assay
(1.80 MB PDF)

Figure S3 SPR analysis
(0.46 MB PDF)

Table S1 All detected reproducible signal peaks
(0.09 MB XLS)

Table S2 Oligonucleotides for PCR
(0.16 MB DOC)

Methods S1 (0.05 MB DOC)
