# Supplementary material for: Use of cDNA Tiling Arrays for Identifying Protein Interactions Selected by In Vitro Display Technologies
Source: PLoS One. 2008 Feb 20;3(2):e1646. doi: 10.1371/journal.pone.0001646 (PMC2241667; doi:10.1371/journal.pone.0001646)

Nrbf2 (NM_025307.2)

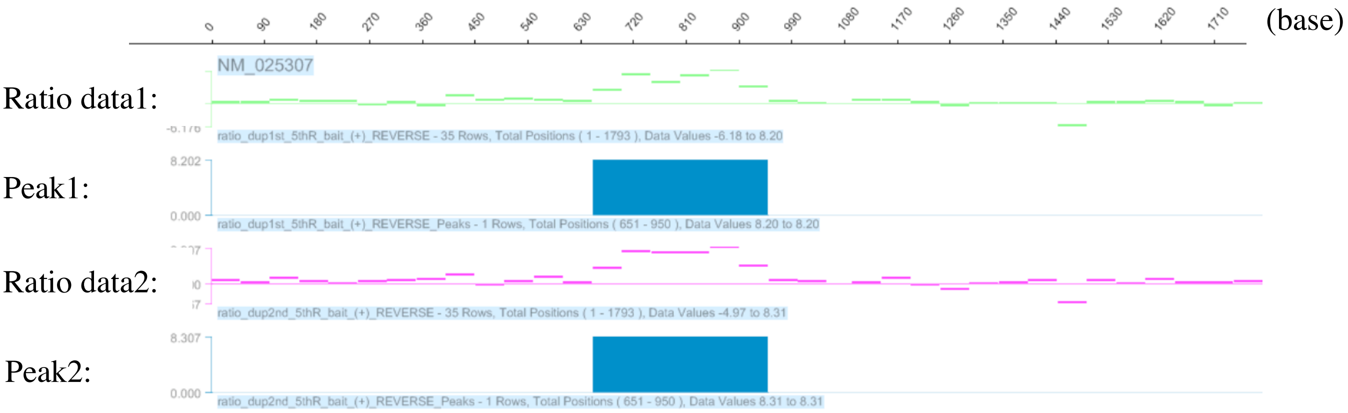

4732436F15Rik (XM_143418.3)

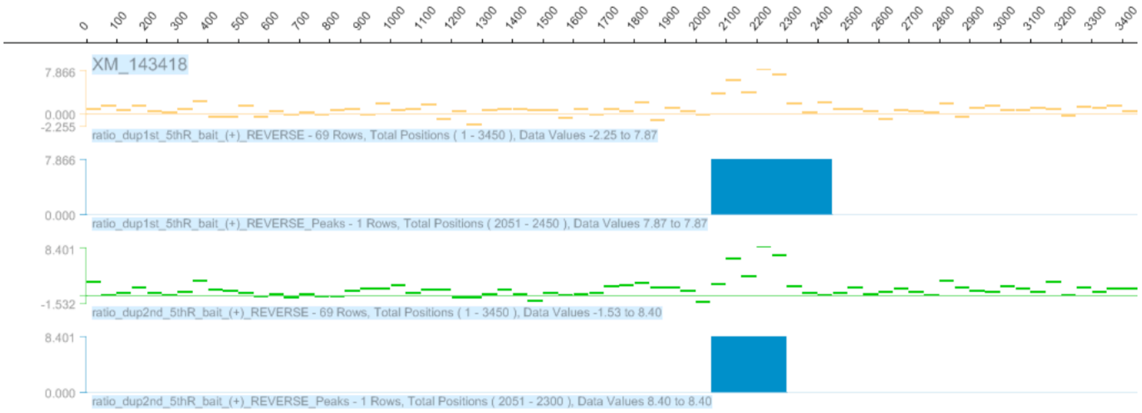

c-Maf (S74567.1)

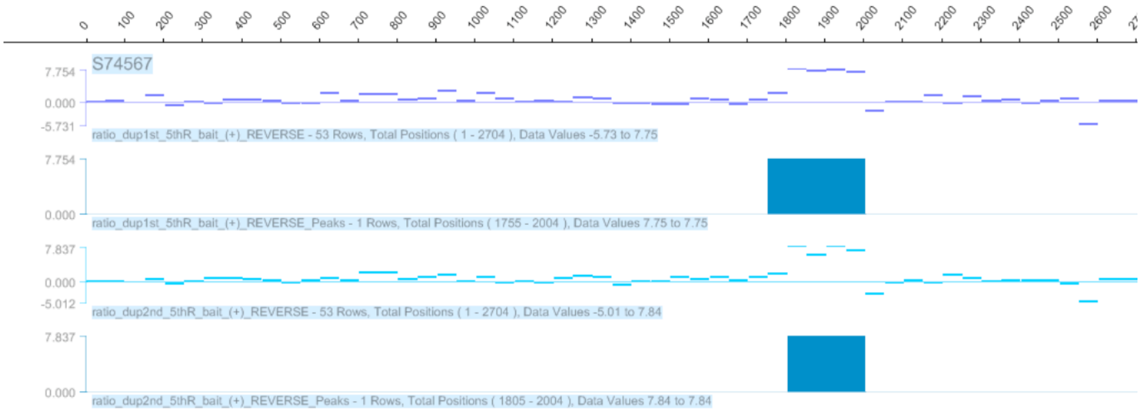

SNAP19 (NM_025925.1)

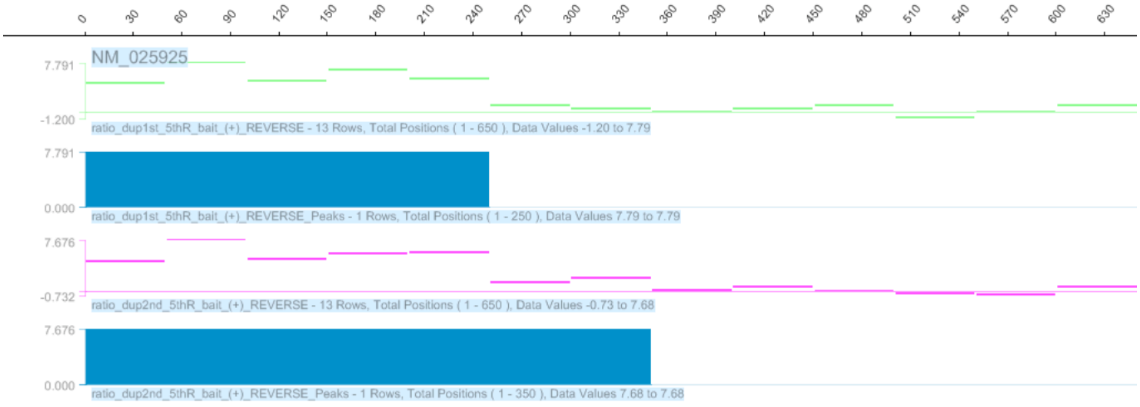

Fos (NM_010234.2)

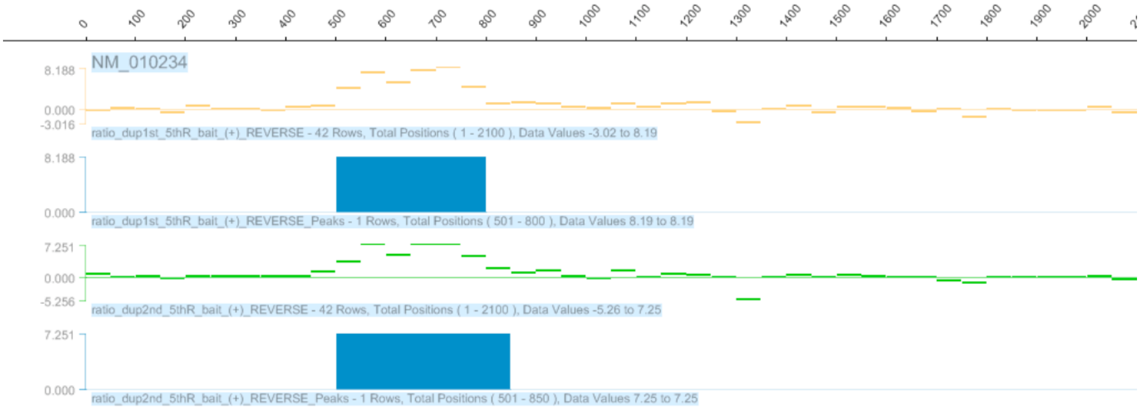

Mapre3 (NM_133350.1)

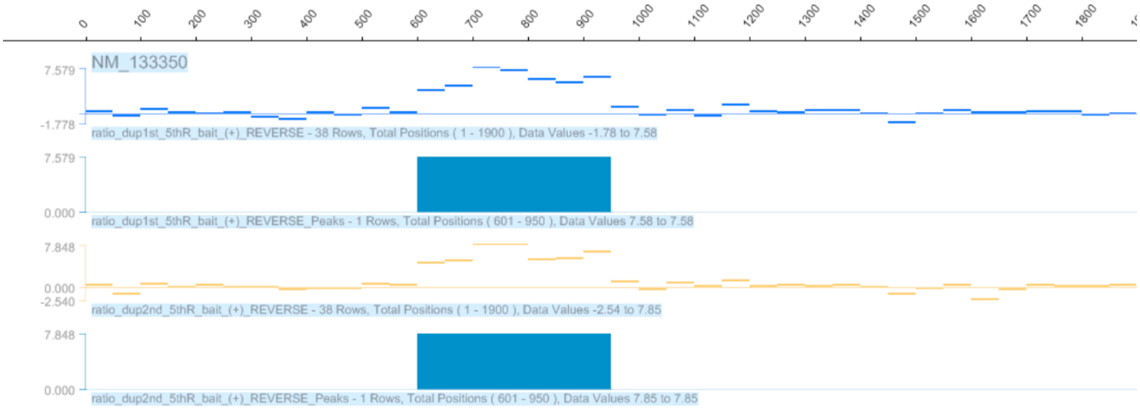

Cspg6 (NM_007790.2)

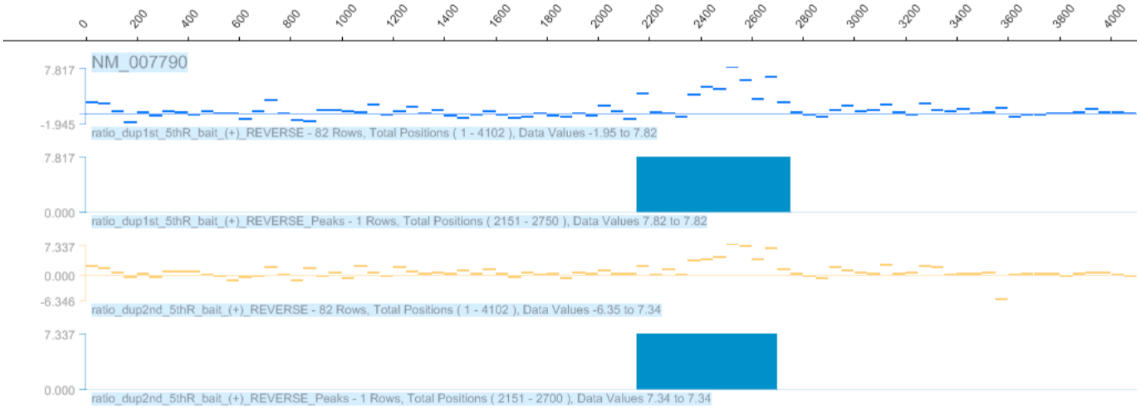

Kif5A (NM_008447.2)

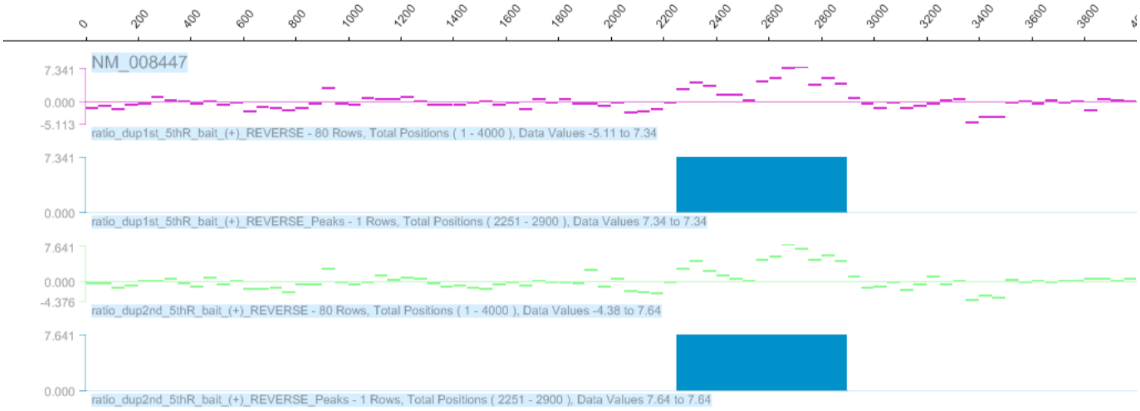

9130229H14Rik (XM_135706.4)

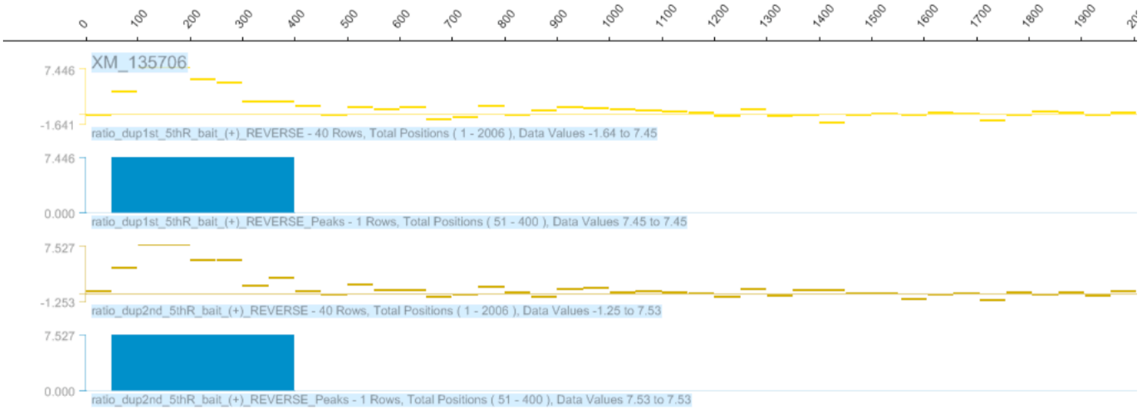

Jun (NM_010591.1)

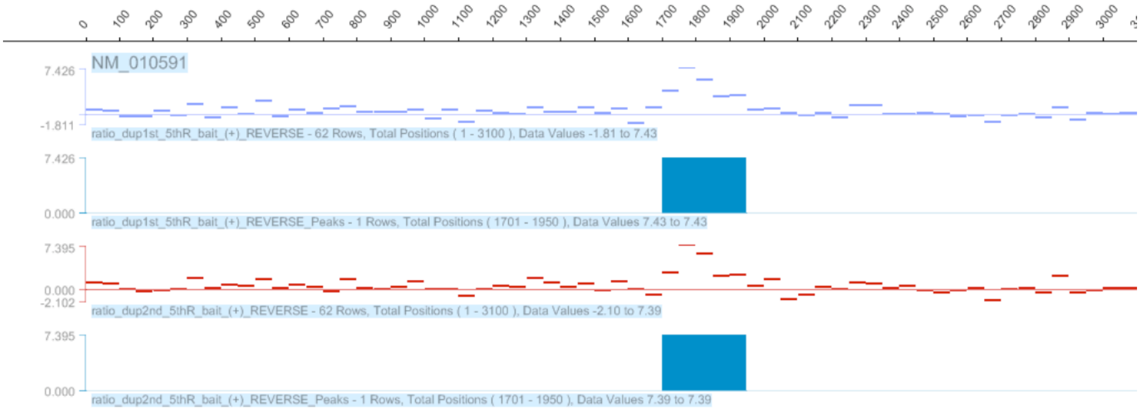

Mapk8ip3 (NM_013931.1)

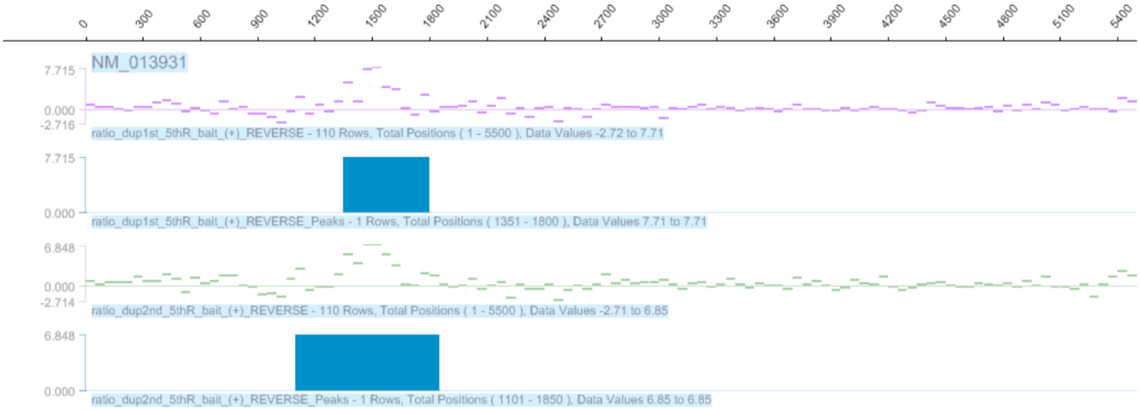

Creb3 (XM_131375.2)

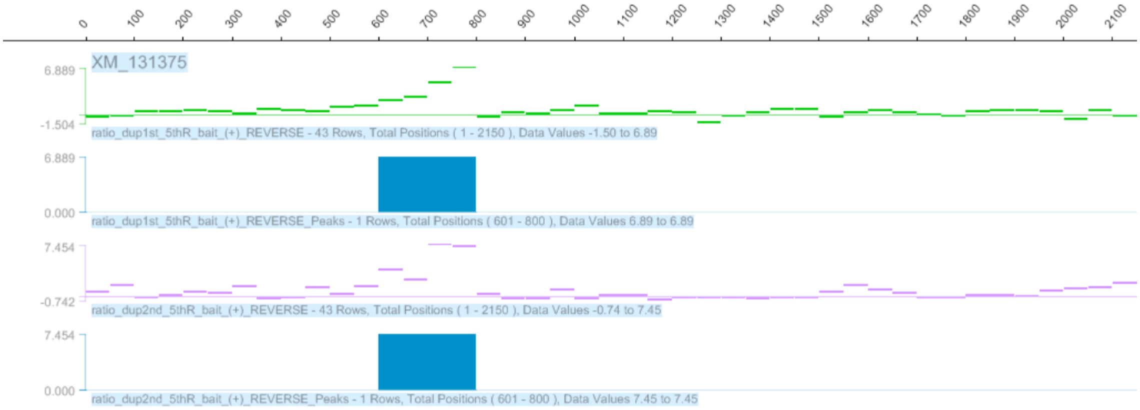

Kif5B (NM_008448.2)

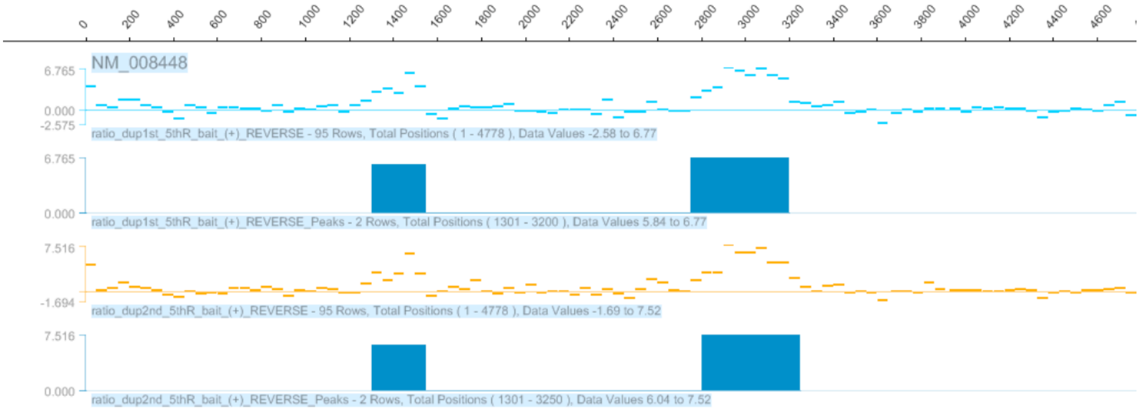

Nef3 (NM_008691.1)

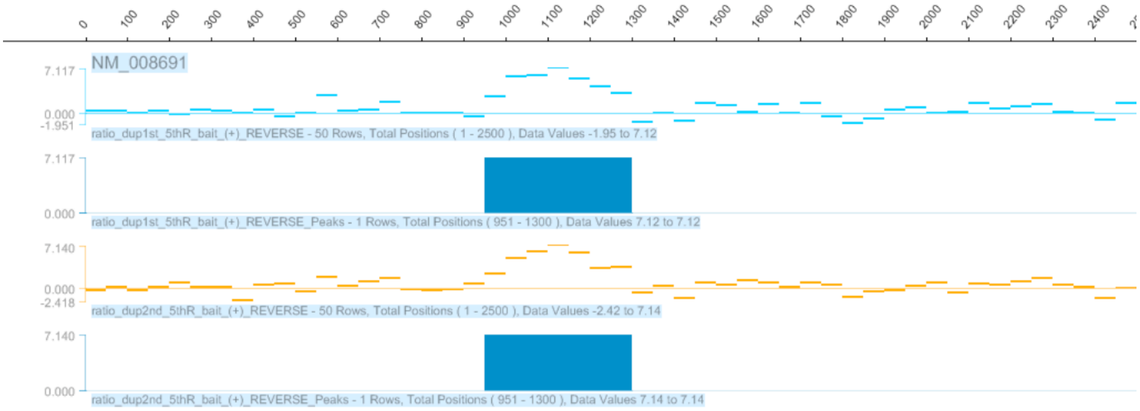

Kif5C (NM_008449.2)

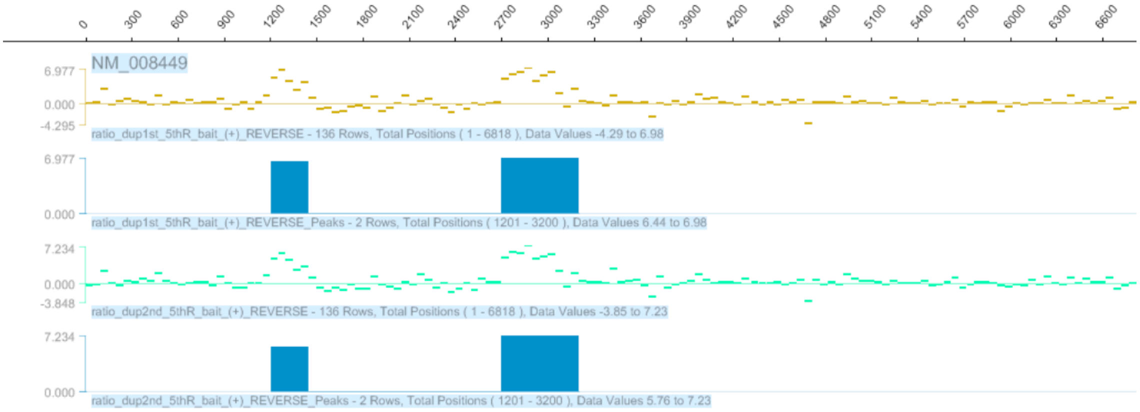

Eef1d (NM_029663.1)

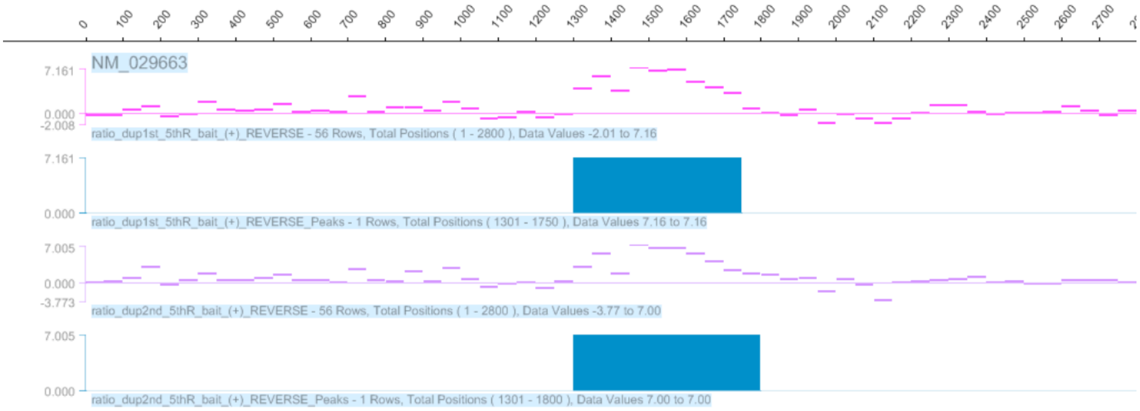

Atf7 (NM_146065.1)

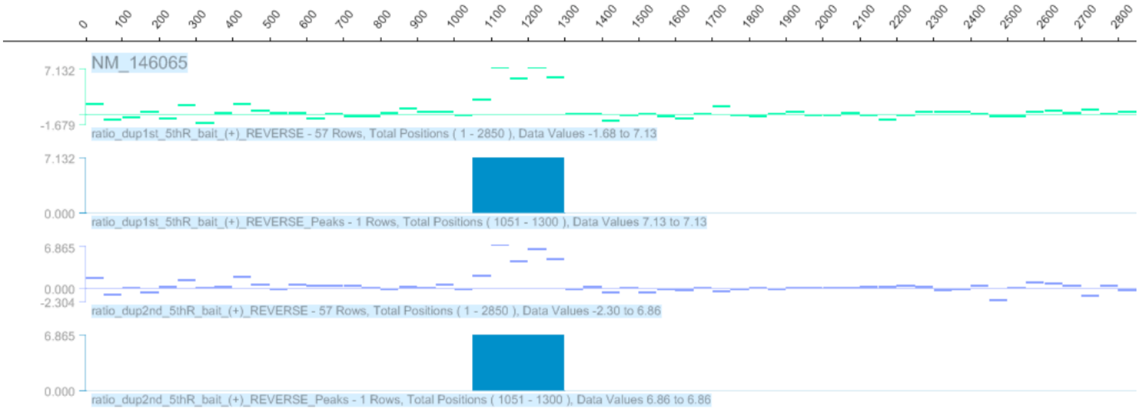

Atf4 (NM_009716.1)

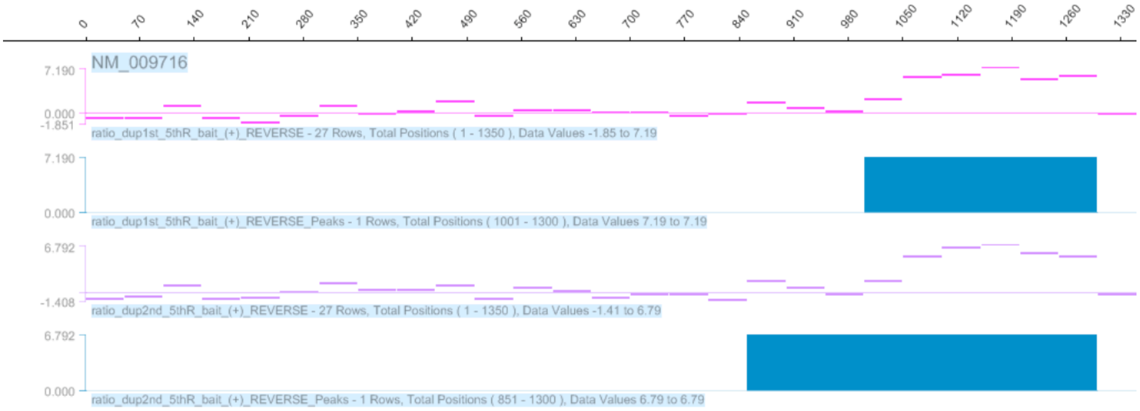

Cutl1 (NM_009986.2)

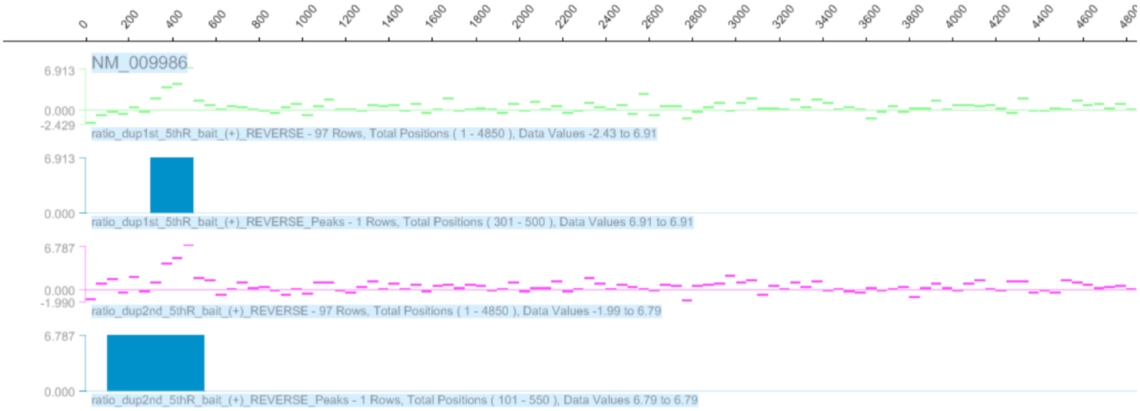

Jdp2 (NM_030887.2)

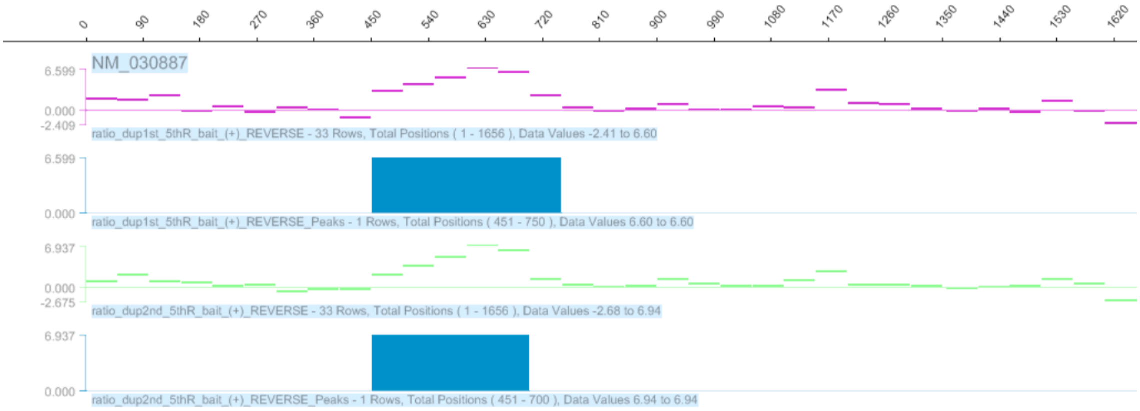

Ofd1 (NM_177429.2)

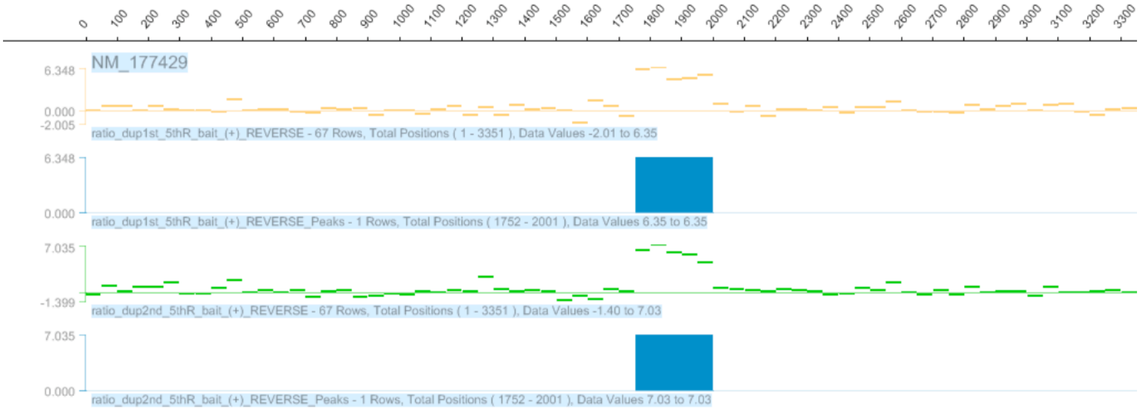

GFAP (NM_010277.1)

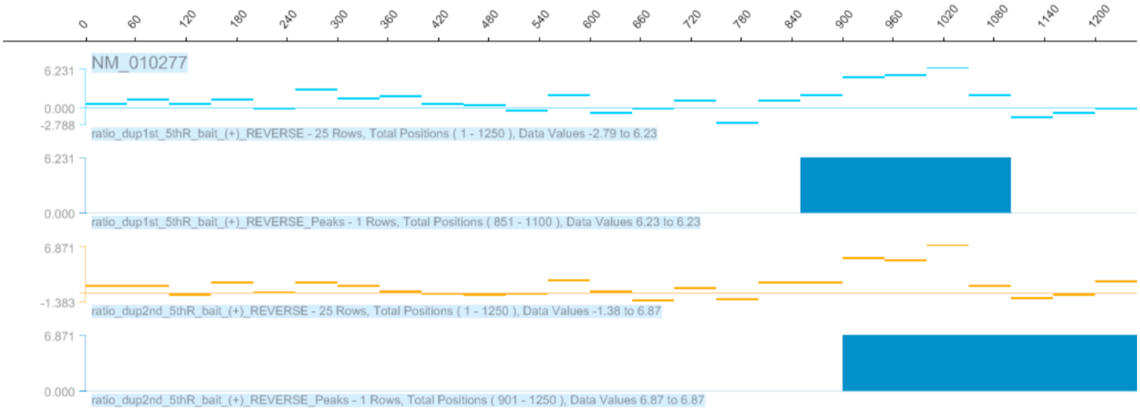

Psmc5 (NM_008950.1)

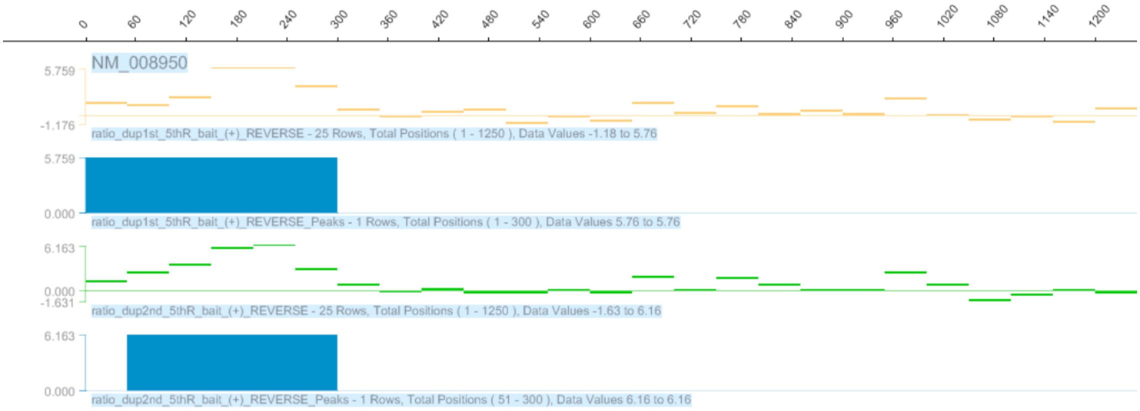

Atf3 (NM_007498.2)

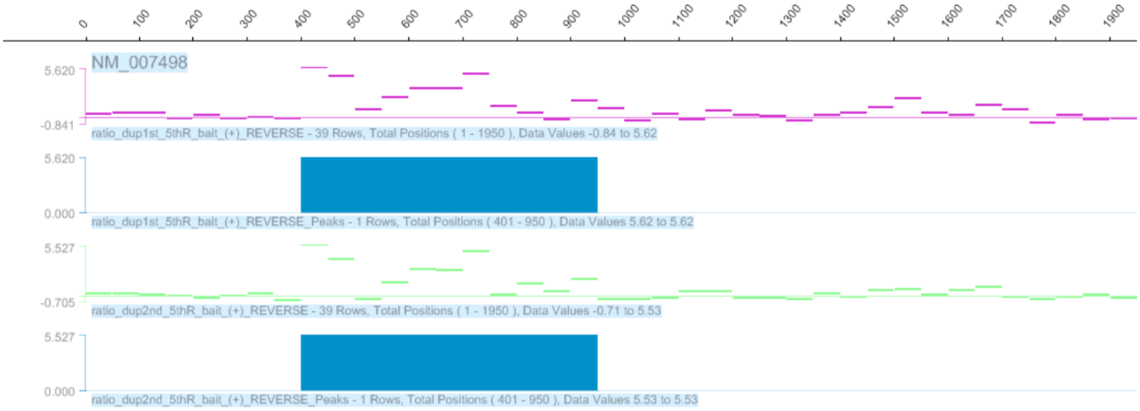

B130050I23Rik (NM_153536.2)

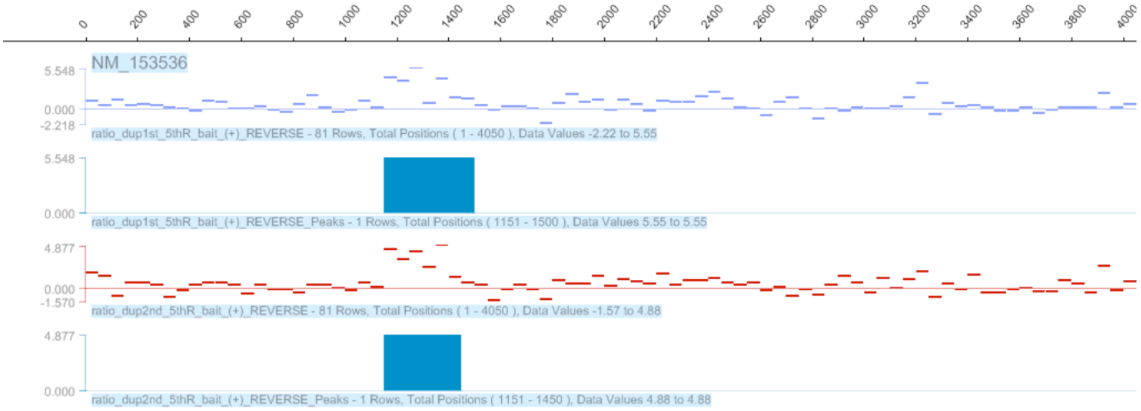

Cebpg (XM_133383.2)

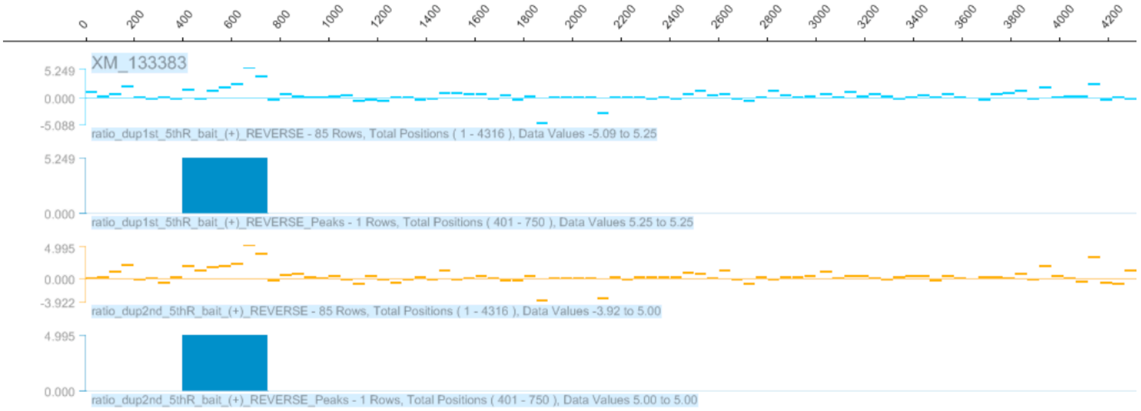

1200008A14Rik (NM_028915.1)

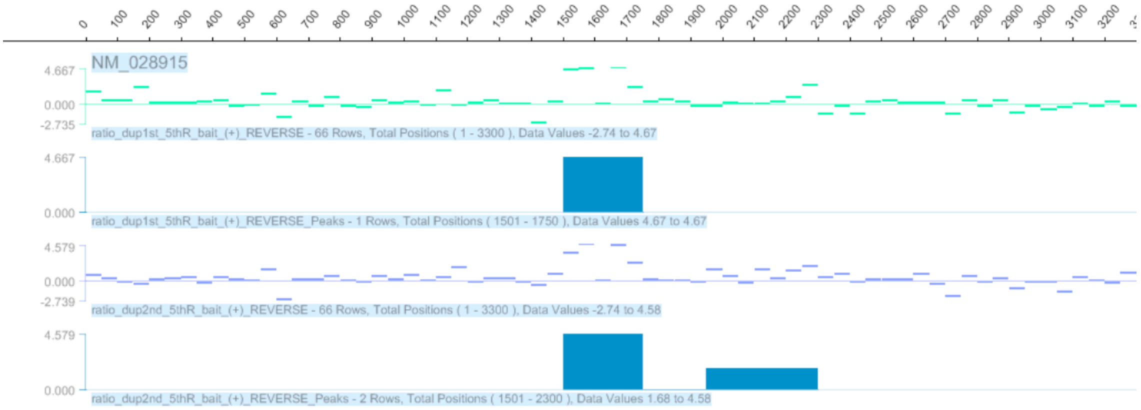

Myh11 (NM_013607.1)

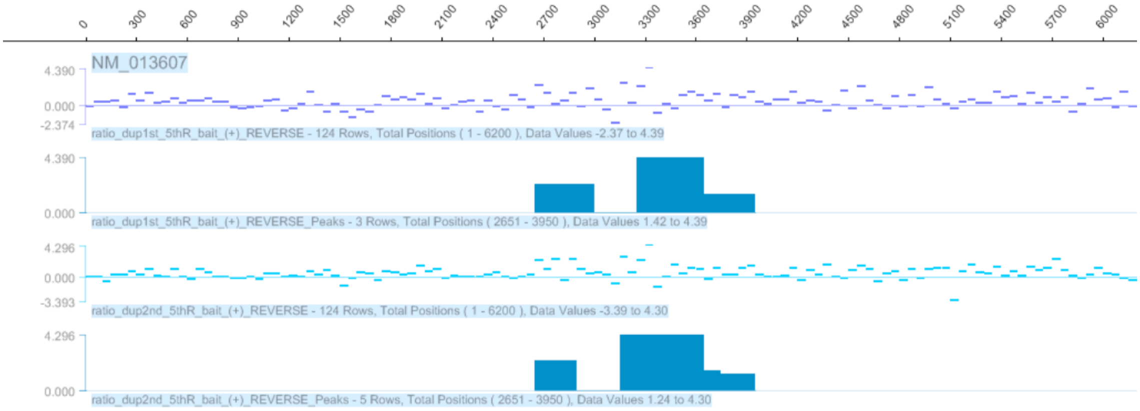

Tax1bp1 (NM_025816.1)

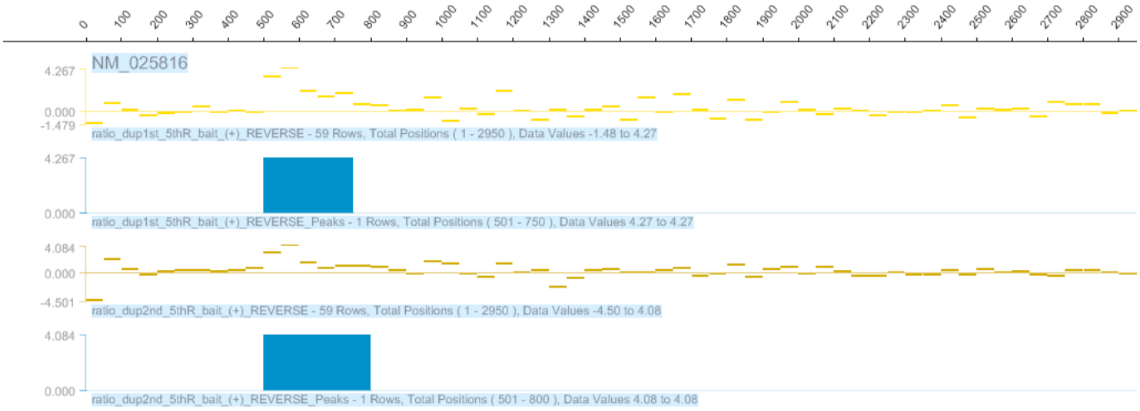

Myt1 (NM_008665.2)

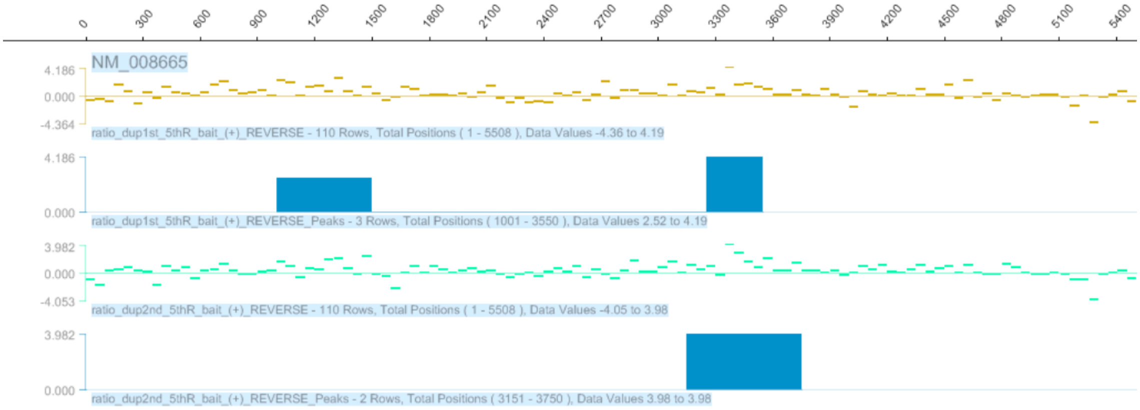

Fosl2 (NM_008037.3)

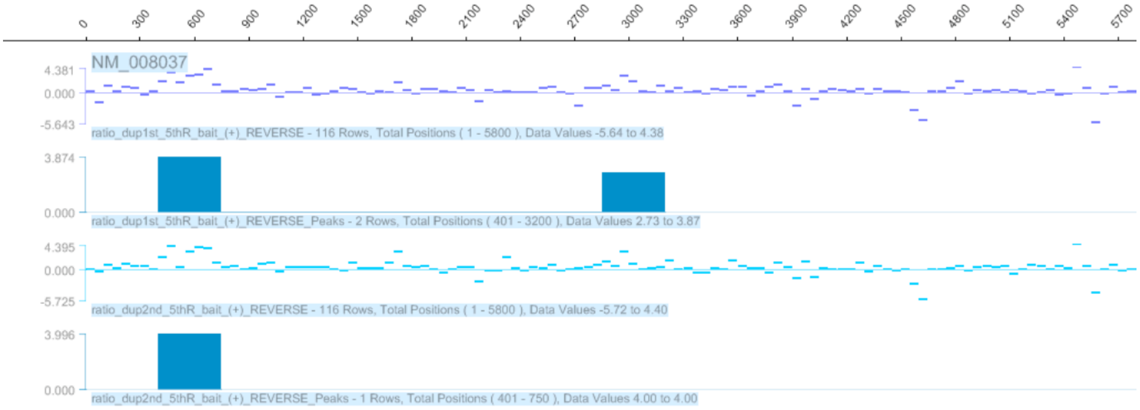

Tef (NM_017376.2)

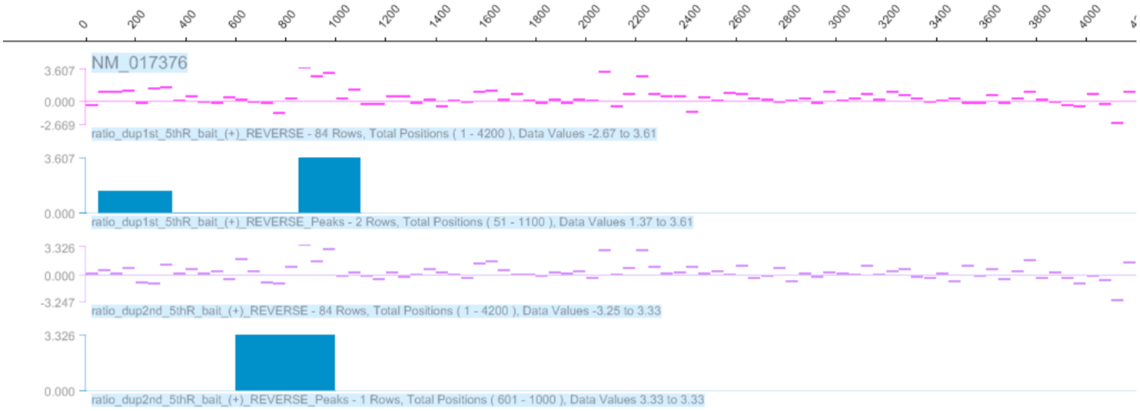

Cebpz (NM_009882.1)

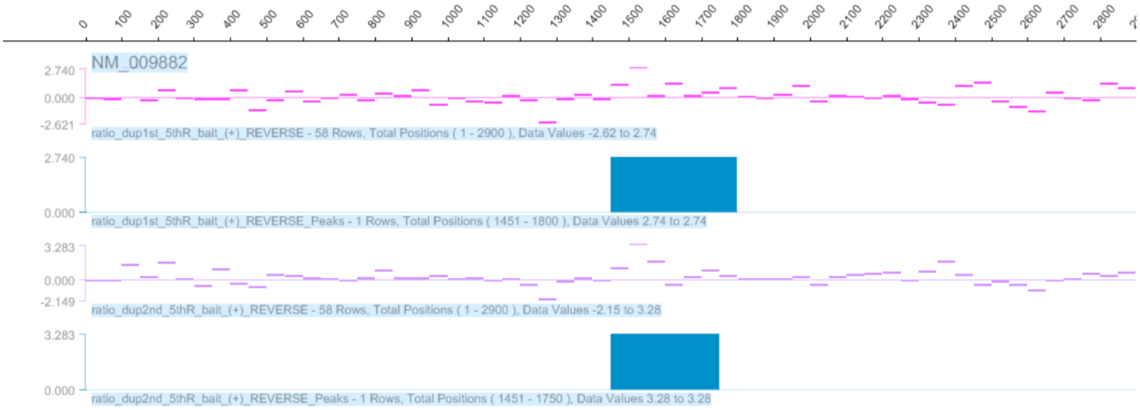

Supplement: Figure S1 — Signal ratio and peak data of selected candidates (2.14 MB PDF) [file pone.0001646.s002.pdf]
