# Supplementary figures and images for: Use of cDNA Tiling Arrays for Identifying Protein Interactions Selected by In Vitro Display Technologies
Source: PLoS One. 2008 Feb 20;3(2):e1646. doi: 10.1371/journal.pone.0001646 (PMC2241667; doi:10.1371/journal.pone.0001646)

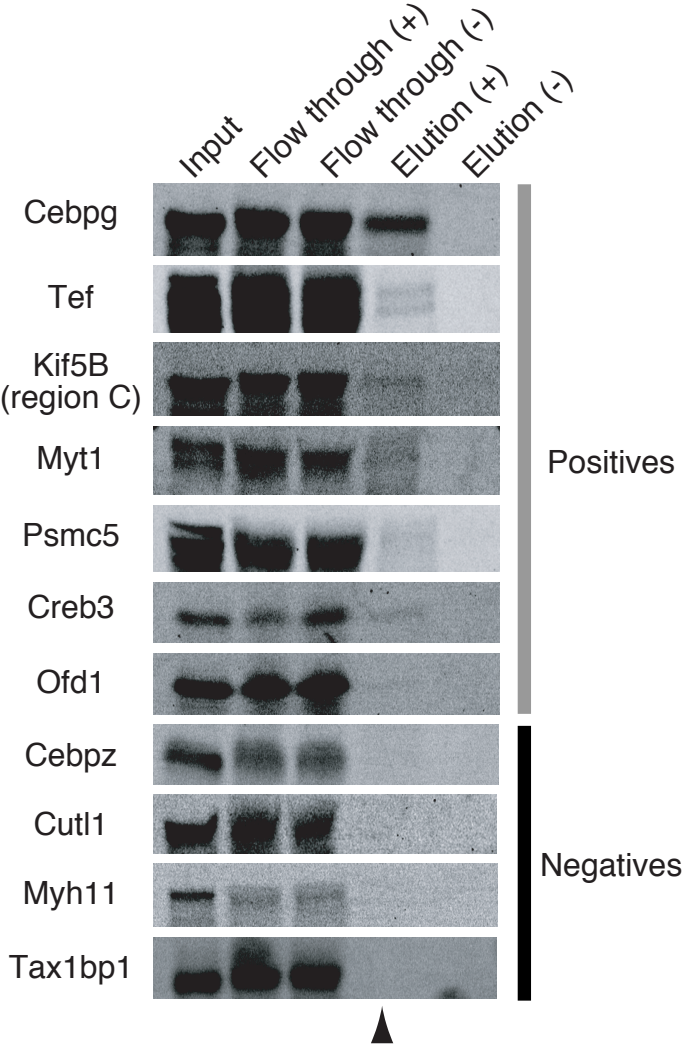

Supplement: Figure S2 — In vitro pull-down assay (1.80 MB PDF) [file pone.0001646.s003.pdf]
