# Supplementary material for: Use of cDNA Tiling Arrays for Identifying Protein Interactions Selected by In Vitro Display Technologies
Source: PLoS One. 2008 Feb 20;3(2):e1646. doi: 10.1371/journal.pone.0001646 (PMC2241667; doi:10.1371/journal.pone.0001646)
Supplement: Figure S3 — SPR analysis (0.46 MB PDF) [file pone.0001646.s004.pdf]

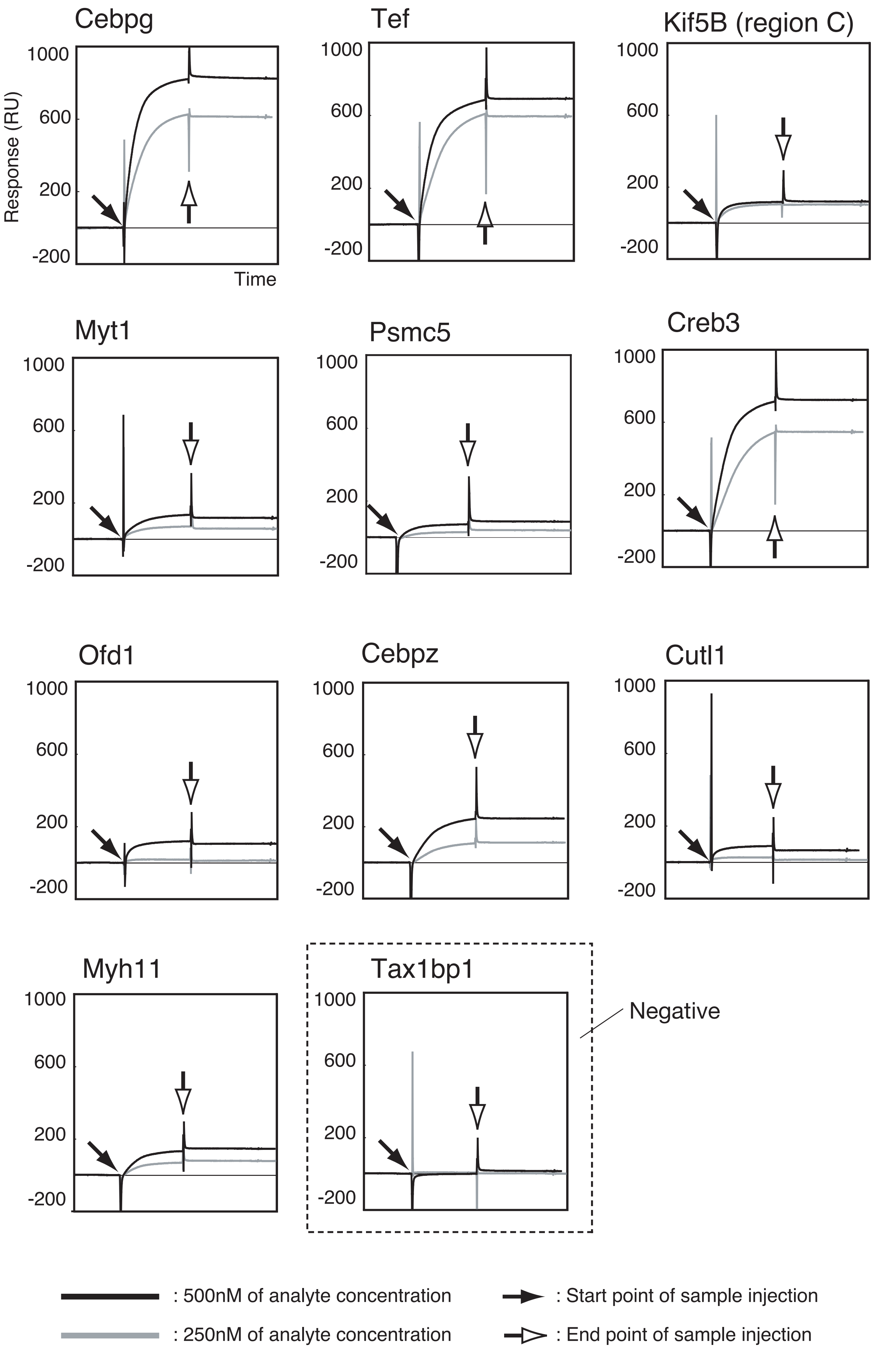
Cebpg
Tef
Kif5B (region C)
Myt1
Psmc5
Creb3
Ofd1
Cebpz
Cutl1
Myh11
Tax1bp1
Negative
Response (RU)
Time
1000
600
200
-200
: 500nM of analyte concentration
: 250nM of analyte concentration
: Start point of sample injection
: End point of sample injection
