# Supplementary material for: Use of cDNA Tiling Arrays for Identifying Protein Interactions Selected by In Vitro Display Technologies
Source: PLoS One. 2008 Feb 20;3(2):e1646. doi: 10.1371/journal.pone.0001646 (PMC2241667; doi:10.1371/journal.pone.0001646)
Supplement: Table S2 — Oligonucleotides for PCR (0.16 MB DOC) [file pone.0001646.s006.doc]

A) Primers for the real-time PCR analysis

| Gene | Name of Primers | Sequence |
| --- | --- | --- |
| Nrbf2  (NM_025307.2) | Nrbf2_F | 5'AGTTCCTTGTGGCTGAGA 3' |
|  | Nrbf2_R | 5'TCCGAGTGTGAAGGTAAG 3' |
| 4732436F15Rik  (XM_143418.3) | 4732436F15Rik_F | 5'AGCTGGAGAAAGAGGTGTCA 3' |
|  | 4732436F15Rik_R | 5'CACAAACTCCCGGTGTAG 3' |
| c-Maf  (S74567.1) | c-Maf_F | 5'AAGAGGGTGCAGCAGAGACA 3' |
|  | c-Maf_R | 5'GTTTTCTCGGAAGCCGTTG 3' |
| SNAP19  (NM_025925.1) | SNAP19_F | 5'AAACCCTGCTGCGTCTAA 3' |
|  | SNAP19_R | 5'ATCATGGATTGAAGGGCTA 3' |
| Fos  (NM_010234.2) | Fos_F | 5'CGAAGGGAACGGAATAAGAT 3' |
|  | Fos_R | 5'GCAACGCAGACTTCTCATC 3' |
| Mapre3  (NM_133350.1) | Mapre3_F | 5'GGACTTGAAGCTGACCGTAG 3' |
|  | Mapre3_R | 5'CAATGATGCCCGAGATGA 3' |
| Cspg6  (NM_007790.2) | Cspg6_F | 5'GCTCTGAATGATGAAATCC 3' |
|  | Cspg6_R | 5'TCTGATGTTGTGGCAGTAAG 3' |
| Kif5A(regionC)  (NM_008447.2) | Kif5A_C_F | 5'CAGCTGGTACGTGACAATGC 3' |
|  | Kif5A_C_R | 5'CCTCCAGGGCCTTAACTCTC 3' |
| 9130229H14Rik  (XM_135706.4) | 9130229H14Rik_F | 5'CCTGGAAGACCAAATCAA 3' |
|  | 9130229H14Rik_R | 5'CATTCAGGATCTCCACTTTC 3' |
| Jun  (NM_010591.1) | Jun_F | 5'ATCGCTCGGCTAGAGGAAA 3' |
|  | Jun_R | 5'CTTAAGCTGTGCCACCTGTT 3' |
| Mapk8ip3  (NM_013931.1) | Mapk8ip3_F | 5'CATTGCTAAGGTTGACCA 3' |
|  | Mapk8ip3_R | 5'GTTACTGCCTCTGACTTGAC 3' |
| Creb3  (XM_131375.2) | Creb3_F | 5'GCGGAGGAAGATTCGTAAC 3' |
|  | Creb3_R | 5'CTCCAAACGCTGCACCTT 3' |
| Kif5B(regionC)  (NM_008448.2) | Kif5B_C_F | 5'AAACAACCTTGAACAGCTC 3' |
|  | Kif5B_C_R | 5'GCTCTAAGCCGTTTCTCTAAC 3' |
| Nef3  (NM_008691.1) | Nef3_F | 5'GGAACCAAGTGGGAAATG 3' |
|  | Nef3_R | 5'CCCTCTAGGAGTTTCCTGT 3' |
| Kif5C(regionC)  (NM_008449.2) | Kif5C_F | 5'TGGTCCGGGACAATGCAG 3' |
|  | Kif5C_R | 5'GGCCTCTTTCAGCGCACTC 3' |
| Eef1d  (NM_029663.1) | Eef1d_F | 5'GAGAACCAGAACCTTCGAG 3' |
|  | Eef1d_R | 5'TCGGGGAGTAGGTGAACT 3' |
| Atf7  (NM_146065.1) | Atf7_F | 5'GGCAAAAGCGGAAACTGT 3' |
|  | Atf7_R | 5'ACTGGGCAATCTTTATGAGC 3' |
| Atf4  (NM_009716.1) | Atf4_F | 5'ACAAGACAGCAGCCACTA 3' |
|  | Atf4_R | 5'CTTACGGACCTCTTCTATCAG 3' |
| Cutl1  (NM_009986.2) | Cutl1_F | 5'CCAGAAACTTAGGGAAACACT 3' |
|  | Cutl1_R | 5'TCTTCAGGGTCTGCTCGTAT 3' |
| Jdp2  (NM_030887.2) | Jdp2_F | 5'TGCAGAGGGAGTCAGAGC 3' |
|  | Jdp2_R | 5'CAGCAGTGGGTTGCCTTC 3' |
| Ofd1  (NM_177429.2) | Ofd1_F | 5'GAAACTCATAGGCAGGCT 3' |
|  | Ofd1_R | 5'CACTTCATTCTCTAGGGC 3' |
| GFAP  (NM_010277.1) | GFAP_F | 5'GGAGGGCCAAAGCCTCAA 3' |
|  | GFAP_R | 5'CCTCCAGCAATTTCCTGTAGGT 3' |
| Kif5C(regionN)  (NM_008449.2) | Kif5C_N_F | 5'GAACTTGGAACTAACAGCAGAA 3' |
|  | Kif5C_N_R | 5'TTCAGCTCCATCTCCAGA 3' |
| Psmc5  (NM_008950.1) | Psmc5_F | 5'CAGGCACAGAGGAATGAGC 3' |
|  | Psmc5_R | 5'CCCTCACGACTTCTCCAAC 3' |
| Kif5B(regionN)  (NM_008448.2) | Kif5B_N_F | 5'CGGAGCAGTGGAAAAAGAAGTA 3' |
|  | Kif5B_N_R | 5'ACGCCAACGGTTTAGCTC 3' |
| Atf3  (NM_007498.2) | Atf3_F | 5'CAAGAAAAAGGAGAAGACAG 3' |
|  | Atf3_R | 5'GCAGGTTGAGCATGTATATC 3' |
| B130050I23Rik  (NM_153536.2) | B130050I23Rik_F | 5'TGGTCCTCAAGGCTTATCA 3' |
|  | B130050I23Rik_R | 5'ATCTGCTCCTCCATCTCTTT 3' |
| Cebpg  (XM_133383.2) | Cebpg_F | 5'GCTCAAGATACACTGCAAAG 3' |
|  | Cebpg_R | 5'CTGTAGTTTCCGTGCTGAT 3' |
| 1200008A14Rik  (NM_028915.1) | 1200008A14Rik_F | 5'AGGCTCTCATTACCACAGAT 3' |
|  | 1200008A14Rik_R | 5'CTCGCGCTTTCATTAATTC 3' |
| Myh11  (NM_013607.1) | Myh11_F | 5'GAAAAGGCTAAAAACCTCAC 3' |
|  | Myh11_F | 5'CAGTTTCCTCTTGAGTTTCT 3' |
| Tax1bp1  (NM_025816.1) | Tax1bp1_F | 5'GGCAATTCCGATATGTTGGT 3' |
|  | Tax1bp1_R | 5'TGGCTCAGTTCTCTCTCCAT 3' |
| Myt1  (NM_008665.2) | Myt1_F | 5'TCTCAGCCCAAAGTTCAAG 3' |
|  | Myt1_R | 5'TTGTTCTCCTCCTCGATG 3' |
| Alx4  (NM_007442.1) | Alx4_F | 5'TCCCACTTGACTCTCCTCTTAG 3' |
|  | Alx4_R | 5'AGGGACAAGAGCCTACCATC 3' |
| Fosl2  (NM_008037.3) | Fosl2_F | 5'TGATCAAGACCATCGGTAC 3' |
|  | Fosl2_R | 5'TGCAGCTTCTCTGTCAGC 3' |
| Tef  (NM_017376.2) | Tef_F | 5'GCCTGAAGGAGAACCAGATCAC 3' |
|  | Tef_R | 5'CCCACCTCCTTGCGAAGC 3' |
| Atbf1  (NM_007496.1) | Atbf1_F | 5'GCTGAAGCTCTTCCAATGTGC 3' |
|  | Atbf1_R | 5'GCCTTGAGCTGCGTGTTGT 3' |
| Cbfa2t1h  (NM_009822.1) | Cbfa2t1h_F | 5'GGACCTCAGGGACAGAAATA 3' |
|  | Cbfa2t1h_R | 5'AGTACAGTGAGGGATCGTCTT 3' |
| Ash2l  (NM_011791.1) | Ash2l_F | 5'GGGGATGCAAACTTGGTTGA 3' |
|  | Ash2l_R | 5'CGCCTGGGTATCCATCACTT 3' |
| Rrad  (NM_019662.1) | Rrad_F | 5'TTACAAGGTACTGCTCCTCG 3' |
|  | Rrad_R | 5'TAGAACGGTCATATGTGTGC 3' |
| Esrra  (NM_007953.1) | Esrra_F | 5'TGCTGCTTACGCTGCCACTC 3' |
|  | Esrra_R | 5'CCCTTGCCTCAGTCCATCAT 3' |
| Myt1l  (NM_008666.1) | Myt1l_F | 5'ACACGTCAGTGGCAGCTTC 3' |
|  | Myt1l_R | 5'GCTCCTTGATCCCTTCATCTAA 3' |
| Max  (NM_008558.1) | Max_F | 5'ACGTAGGGACCACATCAAAG 3' |
|  | Max_R | 5'ATGTCTTGCTGGTGCGTATC 3' |
| Junb  (NM_008416.1) | Junb_F | 5'GGCCACCAAGTGCCGGAA 3' |
|  | Junb_R | 5'CTTAGGAGACCGGCAGCACT 3' |
| Cebpz  (NM_009882.1) | Cebpz_F | 5'AAGGCGTTTGTGAAGAGGTT 3' |
|  | Cebpz_R | 5'GTTTTCTTCGTCAGACTCCG 3' |
| Evpl  (NM_025276.2) | Evpl_1_F | 5'AGACCATGCAACTTCACCTG 3' |
|  | Evpl_1_R | 5'CTTCTCCTTCACGTTCATCTC 3' |
| Neurod2  (NM_010895.2) | Neurod2_F | 5'ACGAGCCGAGGAGCGACAA 3' |
|  | Neurod2_R | 5'GCCAACGTGGGTTCAGGGAT 3' |
| Nr2f1  (NM_010151.1) | Nr2f1_F | 5'GCTGGCGAGATCCGCAGGAC 3' |
|  | Nr2f1_R | 5'AGCCCGCCTGCTGCTGCTC 3' |
| Tead3  (NM_011566.1) | Tead3_F | 5'TGTTCCCAGGTCCCTTAC 3' |
|  | Tead3_R | 5'TAGGGAGGTAGCTGAACCA 3' |
| Hoxb7  (NM_010460.1) | Hoxb7_F | 5'ACTCAAATAAAGGGGCAAAC 3' |
|  | Hoxb7_R | 5'CCTTCCGATACTGTACAAAAAC 3' |
| Ncor2  (NM_011424.1) | Ncor2_F | 5'AGAGAACAATCCGCGAAGG 3' |
|  | Ncor2_R | 5'GCCCACCCTGCTCTGCAT 3' |
| Pitx1  (NM_011097.1) | Pitx1_F | 5'CACCACCACCGCACGACA 3' |
|  | Pitx1_R | 5'TCCTCTGGCCCCTTGGCTTC 3' |
| Crx  (NM_007770.2) | Crx_F | 5'CCAGTACCCGGATGTGTATG 3' |
|  | Crx_R | 5'AGGACGAGCCTTGGTCTGT 3' |
| Evpl  (NM_025276.2) | Evpl_2_F | 5'ACGGAGGATGCTGTGTACG 3' |
|  | Evpl_2_R | 5'CTTCGGGTCCTTCTGGGT 3' |
| Shox2  (NM_013665.1) | Shox2_F | 5'CGTCCGGGAGCTGGACAT 3' |
|  | Shox2_R | 5'GTCTGGCCTTCGTCCTCCAT 3' |
| Mapkapk2  (XM_129464.2) | Mapkapk2_F | 5'GGACAGTCTTCGCACCAAA 3' |
|  | Mapkapk2_R | 5'GACCATGGCACAAAGACTCT 3' |
| Bapx1  (NM_007524.1) | Bapx1_F | 5'GCGAGGATGACAGCGTTA 3' |
|  | Bapx1_R | 5'CTTCTTTCGCGGTTTAGG 3' |
| Map3k14  (NM_016896.2) | Map3k14_F | 5'GCCTGGCTAAGACATGGT 3' |
|  | Map3k14_R | 5'TCATTCTGTGGACCTCGC 3' |
| Arf4l  (NM_031160.1) | Arf4l_F | 5'TAGGGGCTGCAAGACCGGAGAT 3' |
|  | Arf4l_R | 5'GCAACTGTGCGAGAGGGAAGA 3' |
| Carhsp1  (NM_025821.2) | Carhsp1_F | 5'CCTGACATCTTCCTGCACAT 3' |
|  | Carhsp1_R | 5'CCTGCAGCTTCTCGTTCTT 3' |
| Luzp1  (NM_024452.1) | Luzp1_F | 5'AAACCTTACGCGAGAGCTA 3' |
|  | Luzp1_R | 5'TCACTTTGACTCTGAGCATT 3' |
| Tbx18  (NM_023814.2) | Tbx18_F | 5'CGGTGGAAGACGCAGGCTG 3' |
|  | Tbx18_R | 5'ACAGTTCCGCTCCGCGTTCG 3' |
| Sh2d3c  (NM_013781.2) | Sh2d3c_F | 5'AGAAACAACAGGCTGTCC 3' |
|  | Sh2d3c_R | 5'TTCTACTGCTGAAGGTGC 3' |
| Nkx3-1  (NM_010921.1) | Nkx3-1_F | 5'CTTGGGTCTCCTGTTGGCCT 3' |
|  | Nkx3-1_R | 5'TCCTTGGACTGGCACATCAC 3' |
| Csf1r  (NM_007779.1) | Csf1r_F | 5'ACCTACGTGTGCAAGACCA 3' |
|  | Csf1r_R | 5'CAGCTTGCTAGGCTCCAA 3' |
| JunD  (NM_010592) | JunD_F | 5'GAGAAAGTCAAGACCCTCAAAAGC 3' |
|  | JunD_R | 5'AGCTGCGCCACCTGCTC 3' |
| Fra1  (NM_010235) | Fra1_F | 5'AGGCGGAGACCGACAAAT 3' |
|  | Fra1_R | 5'CTGCTTCTGCAGCTCTTCA 3' |
| Batf  (NM_016767) | Batf_F | 5'AGTGAGGACCTGGAGAAAC 3' |
|  | Batf_R | 5'GCTCAGCACTGATGTGAAGTA 3' |
| MafA  (NM_194350) | MafA_F | 5'CGCAGTCGTGCCGCTTCAAG 3' |
|  | MafA_R | 5'TACAGGTCCCGCTCCTTGGC 3' |
| MafG  (NM_010756) | MafG_F | 5'AGGCGCACACTGAAGAAC 3' |
|  | MafG_R | 5'CTCCGCCTTCTGCTTCTC 3' |
| Nrl  (NM_008736) | Nrl_F | 5'CGCTCAGGCTTGTCGCTC 3' |
|  | Nrl_R | 5'GGTCACAGCGGGCCTTATAG 3' |
| Nrf1  (NM_010938) | Nrf1_F | 5'GCCAATGTCCGCAGTGAT 3' |
|  | Nrf1_R | 5'ACGGTCTGTGATGGTACGAG 3' |
| Nrf2  (NM_010902) | Nrf2_F | 5'GGCCACTTAAAAGACGAGA 3' |
|  | Nrf2_R | 5'GACTTCAAGATACAAGGTGCT 3' |
| NF-IL6  (NM_009883) | NF-IL6_F | 5'ACGAGCGGCTGCAGAAGA 3' |
|  | NF-IL6_R | 5'AAGTTCCGCAGGGTGCTGA 3' |
| CHOP  (NM_007837) | CHOP_F | 5'CGGCTCAAGCAGGAAATC 3' |
|  | CHOP_R | 5'ACCATGCGGTCGATCAGA 3' |
| Elf-1  (NM_007920) | Elf-1_F | 5'AGCTACCTGTCCTAAGTACATC 3' |
|  | Elf-1_R | 5'ACAATCTAGACACGGCTTTA 3' |
| PU.1  (NM_011355) | PU.1_F | 5'TGGGTGGACAAGGACAAA 3' |
|  | PU.1_R | 5'CCATCTTCTGGTAGGTCATCT 3' |
| Ets-1  (NM_011808) | Ets-1_F | 5'AGTCTTGTCAGTCCTTTATCAGC 3' |
|  | Ets-1_R | 5'TCGCACACAAAGCGGTAT 3' |
| Ets-2  (NM_011809) | Ets-2_F | 5'AGTCTTGTCAGTCCTTTATCAGC 3' |
|  | Ets-2_R | 5'TCGCACACAAAGCGGTAT 3' |
| Fli-1  (NM_008026) | Fli-1_F | 5'CCAACGGGGAGTTCAAAATG 3' |
|  | Fli-1_R | 5'CTTTTGCCATGCACTTTGGT 3' |
| Erg  (NM_133659) | Erg_F | 5'CAGACAGCTCCAACTCCAAC 3' |
|  | Erg_R | 5'TTGGGCTTGCTCTTCCTCT 3' |
| SMAD3  (NM_016769) | SMAD3_F | 5'GAAGGCGGTCAAGAGCTT 3' |
|  | SMAD3_R | 5'GCACTTGGTGTTCACGTTCT 3' |
| NFAT1  (NM_010899) | NFAT1_F | 5'CCACGGCTACATGGAGAACA 3' |
|  | NFAT1_R | 5'AGGCGTGCGGCTTAAGGAT 3' |
| NFAT3  (NM_023699) | NFAT3_F | 5'ATTGGCACTGCAGATGAG 3' |
|  | NFAT3_R | 5'GGTACCACTGACTACAGCTTC 3' |
| NFAT4  (NM_010901) | NFAT4_F | 5'GGAACAGCCGATGATAGATA 3' |
|  | NFAT4_R | 5'GTAGAAGTGGGATTTCCAGA 3' |
| Jab1  (NM_013715) | Jab1_F | 5'TTGATGCTCGGGAAAGTC 3' |
|  | Jab1_R | 5'GGCTATGATACCAACCGATT 3' |
| Atf2  (NM_009715) | Atf2_F | 5'GCTTCAAGATGCCGACAA 3' |
|  | Atf2_R | 5'TTCATTTCTCAGCAGGGTG 3' |

B) Primers for the constructions for *in vitro* pull-down assay

| Gene | NameofPrimers | Sequence |
| --- | --- | --- |
| Creb3 | 5’ Creb3 (for cloning) | 5'CACCCCCTCAACCCTTCCTCTCAC 3' |
|  | 3’ Creb3 (for cloning) | 5'TTAGGTTTTGTTGGCTATCTCAATCACC 3' |
|  | 5’ DirecT7_Creb3 (for template construction) | 5'GACAGCAAATGGCGAATTCCCCCTCAACCCTTCCTCTCAC 3' |
|  | 3’ DirectFXA_Creb3 (for template construction) | 5'TCGTCATCGTCCTTGTAGTCAAGCTTGGTTTTGTTGGCTATCTCAATCACC 3' |
| Kif5B  (regionC) | 5’ Kif5B_C (for cloning) | 5'CACCACAGACCAAGAGAAGAGCAGGAAG 3' |
|  | 3’ Kif5B_C (for cloning) | 5'TTACGACTGCTTGCCTCCACC 3' |
|  | 5’ DirecT7_Kif5B_C  (for template construction) | 5'GACAGCAAATGGCGAATTCCAAGCTCTTTGTTCAGGACTTGGCTAC 3' |
|  | 3’ DirectFXA_Kif5B_C  (for template construction) | 5'TCGTCATCGTCCTTGTAGTCAAGCTTAACTGTGCCCGGGTGAGTTG 3' |
| Ofd1 | 5’ Ofd1 (for cloning) | 5'CACCGATGGTTTTCCTCACCGTTCCAAG 3' |
|  | 3’ Ofd1 (for cloning) | 5'TTAAGACTCTGGGGAACTAGCTTC 3' |
|  | 5’ DirecT7_Ofd1  (for template construction) | 5'GACAGCAAATGGCGAATTCCGAACTGGAGCACAAGGACTTTGA 3' |
|  | 3’ DirectFXA_Ofd1  (for template construction) | 5'TCGTCATCGTCCTTGTAGTCAAGCTTCATCTTGCCGCTCAATAGGAGG 3' |
| Cutl1 | 5’ Cutl1 | 5'CACCATGTTGTGCGTAGCCGGAGCCAAG 3' |
|  | 3’ Cutl1 | 5'TTAATCTGGAGCCTTCTGGATCTGCG 3' |
| Psmc5 | 5’ Psmc5 | 5'CACCATGGCGCTTGATGGGCCAG 3' |
|  | 3’ Psmc5 | 5'TCACTTCCATAGCTTCTTGATGGACATGTT 3' |
| Cebpg | 5’ Cebpg | 5'CACCATGAGCAAGCTGTCGCAGCC 3' |
|  | 3’ Cebpg | 5'CTACTGCCCTGGGTTATCAGAATTTG 3' |
| Myh11 | 5’ Myh11 | 5'CACCGCGAACGCCATCCCCAAAGG 3' |
|  | 3’ Myh11 | 5'TTAAGCTGTGCTGTCCAGCGTATC 3' |
| Tax1bp1 | 5’ Tax1bp1 | 5'CACCATGACTTCCTTTCAAGAAGTCCAATTG 3' |
|  | 3’ Tax1bp1 | 5'CTAGTCGAAGTTGAGAACATTCTGATC 3' |
| Myt1 | 5’ Myt1 | 5'CACCGAGCGGAAGTATCCAGGGGAAG 3' |
|  | 3’ Myt1 | 5'CTAGACCTGAATGCCCCTCACAGCTTG 3' |
| Tef | 5’ Tef | 5'CACCATGTCCGACGCGGGCGGCGGGAAGAAG 3' |
|  | 3’ Tef | 5'TTACAAGGGCCCGTACTTGGTCTCGTACTTGGAC 3' |
| Cebpz | 5’ Cebpz | 5'CACCTTCCGCTCAAATATCAGCCCCAAAG 3' |
|  | 3’ Cebpz | 5'TTATGTATTTTCAGCTCCGCAGAATAAAG 3' |
