# Supplementary material for: Use of cDNA Tiling Arrays for Identifying Protein Interactions Selected by In Vitro Display Technologies
Source: PLoS One. 2008 Feb 20;3(2):e1646. doi: 10.1371/journal.pone.0001646 (PMC2241667; doi:10.1371/journal.pone.0001646)
Supplement: Methods S1 — (0.05 MB DOC) [file pone.0001646.s007.doc]

# Calculation of abundance of cDNA fragments

The percentage of cDNA fragments derived from an mRNA expressed as a single copy in a cell was calculated as follows.

Percentage of cDNA fragments = (C / AB) × 100 (%)

Parameters (Campbell 1996):

(A) Number of mRNA molecules per cell: 2 × 10^5^ ~ 1 × 10^6^ molecules/cell

(B) Average size of mRNA molecules: 1,900 bases

(C) Average size of the resulting cDNAs from the IVV screening: 225 bp

# Preparation of bait and IVV library template RNAs

The cDNA of mouse Jun (167-319 amino acids) was amplified by PCR using primers containing a BamHI site or an XhoI site, and the product was digested with BamHI and XhoI. The fragment was sub-cloned into the BamHI/XhoI site of pCMV-CBPzz vector, which contains an SP6 promoter, a part of tobacco mosaic virus omega enhancer (O’ sequence), an N-terminal T7-tag coding sequence and a C-terminal TAP tag coding sequence (Horisawa et al. 2004). From the resulting plasmid, a bait template DNA was amplified by PCR, and the PCR product was purified with a QIAquick PCR purification kit (Qiagen). The purified DNA was used as a template for *in vitro* transcription with a RiboMax large-scale RNA purification system-SP6 (Promega). The RNA was purified with an RNeasy mini kit (Qiagen).

As a source for the IVV library, we choose poly (A)+ RNA derived from whole brains pooled from 200 male/female BALB/c mice, 8-12 weeks of age (BD Bioscience). The RNA was primed using the oligonucleotide 5’-TCGTCATCGTCCTTGTAGTCAAGCTTN_9_-3’ and cDNA was synthesized using a SuperScript double strand cDNA synthesis kit (Invitrogen). The cDNA was ligated with adaptor DNA (forward oligonucleotide: 5’-TAGCATGACTGGTGGACAGCAAATGGGTGCGGCCGCGAATTCC-3’; reverse oligonucleotide: 5’-GGAATTCG-3’) using a Ligation High kit (Toyobo). After ethanol precipitation, the ligated DNA was PCR-amplified with primers, 5’F3 (5’-GGAAGATCTATTTAGGTGACACTATAGAACAACAACAACAACAAACAACAACAAAATG-3’) and 3’lib_PCR (5’-TTTTTTTTCTTGTCGTCATCGTCCTTGTAGTC-3’). The PCR product was purified with the QIAquick kit and fractionated (over ~200 bp) with a CHROMA SPIN-1000 (BD Bioscience). The fractionated PCR product was used as a template for transcription using the RiboMax large RNA production system-SP6. The resulting RNA was ligated with PEG Puro spacer [p(dCp)2-T(Fluor)p-PEGp-(dCp)2-puromycin] (Miyamoto-Sato et al. 2003) using T4 RNA ligase (Takara). The ligated RNA was purified with the RNeasy mini kit

# Affinity screening

A 50 µl aliquot of wheat germ extract reaction mixture (Promega) containing 10 pmol of the bait Jun RNA, 10 pmol of the ligated library RNA, 80 µM amino acid mixture, 76 mM potassium acetate and 40 U of RNase inhibitor (Invitrogen) was incubated for 1 h at 26˚C. Subsequently, the reaction mixture was added to 50 µl of rabbit IgG agarose beads (Sigma) equilibrated with 50 µl of IPP150 buffer (10 mM Tris-HCl, pH 8.0, 150 mM NaCl and 0.1% NP-40), and mixed on a rotator for 2 h at 4˚C. The beads were washed with 800 µl of IPP150 once and TEV cleavage buffer (IPP150 plus 0.5 mM EDTA and 1 mM DTT) five times, then 100 µl of TEV cleavage buffer containing 100 U of TEV protease (Invitrogen) was added, and rotation was continued for 2 h at 16˚C. The resulting eluate was used as the RT-PCT template. RT-PCR was performed with a OneStep RT-PCR kit (Qiagen) using primers 5’F3 and 3’Flag-1AL (5’-TTTTTTTTCTTGTCGTCATCGTCCTTGTAG-3’). The RT-PCR product was used for the next round of selection as described. After five rounds of screening, the RT-PCR product was analyzed by using a tiling array.

# Microarray experiment

After the iterative selection, *in vitro* transcription was employed in order to produce biotin-labeled RNA from the screened cDNA library using a MEGAscript SP6 kit (Ambion). Briefly, 1 µg of double-stranded cDNA was incubated with 7.5 mM ATP and GTP, 5.6 mM UTP and CTP, 1.9 mM bio-11-CTP and bio-16 UTP (Enzo) and 1x SP6 enzyme mix in 1x transcription buffer for 5 h at 37˚C. The RNA was purified using a RNeasy RNA purification kit (Qiagen) according to the manufacturer’s instructions. The quality of the RNA was monitored with a Bioanalyzer (Agilent).

Microarrays were hybridized with 12 µg of RNA in 300 µl, in the presence of 50 mM MES, 0.5 M NaCl, 10 mM EDTA and 0.005% (v/v) Tween-20 for 16 h at 45˚C. Before application to the array, samples were heated to 95˚C for 5 min, heated to 45˚C for 5 min, and spun at 14,000 g for 5 min. After hybridization, arrays were washed in non-stringent buffer [6x SSPE, 0.01% (v/v) Tween-20] for 5 min at room temperature, followed by washing in stringent buffer (100 mM MES, 0.1 M NaCl, 0.01% Tween-20) for 30 min at 45 ˚C. After washing, arrays were stained with streptavidin-Cy3 conjugate (Amersham) for 25 min at room temperature, followed by washing with non-stringent buffer, a 30 sec rinse in 1x final rinse buffer (Nimblegen), and a drying step.

After hybridization, the glass slides were scanned with a GenePix 4000B scanner (Axon) and GenePix software (Axon). Scanned images were analyzed using Nimlegen Scan software (Nimblegen). All procedures described above were carried out by Genefrontier Co. and NimbleGen Systems Inc.

***In vitro* pull-down assay**

In the prey DNA templates, Psmc5, Cebpg and Tax1bp1 were PCR-amplified from FANTOM clone plasmids (Dnaform), and others were cloned from the IVV template cDNA library by PCR with the primers indicated in **Supplementary Table 2** online. Amplified cDNA fragments were cloned into pENTR D-TOPO vector by using a pENTR Directional TOPO Cloning Kit (Invitrogen) and subsequently recombined into pcDNA3.1/mV5-DEST vector (Invitrogen) by using Gateway LR Clonase Enzyme Mix (Invitrogen) according to the manufacturer’s instructions. Three of the 11 recombined clones, Creb3, Kif5B (region C) and Ofd1, were amplified by tandem PCR with the primers for template construction indicated in **Supplementary Table 2** online and the following common primer set (forward oligonucleotide: 5’- GGAAGATCTATTTAGGTGACACTATAGAACAACAACAACAACAAACAACAACAAAATGGCTAGCATGACTGGTGGACAGCAAATGGCGAATTCC-3’; reverse oligonucleotide: 5’-TTTTTTTTCTCGAGCTTGTCGTCATCGTCCTTGTAGTC-3’). Others were also amplified by PCR with another primer set (forward oligonucleotide: 5’- ATTTAGGTGACACTATAGAACAACAACAACAACAAACAACAACAAAATGGCTAGCATGGGTAAGCCTATCCCTAACCCTC-3’; reverse oligonucleotide: 5’- ACCCTAATCAAGTTTTTTGGGGTCGAGG-3’). The PCR products were transcribed by MEGAscript SP6 kit (Ambion). On the other hand, the bait template RNA was the same as used in the IVV screening. The bait and preys were translated separately in a wheat germ extract cell-free translation system (Zoegene). Only preys were fluorescence-labeled, as described in our previous report (Horisawa et al. 2004). The bait and preys in IPP150 buffer were incubated with Magnotex SA beads (Takara) conjugating Biotin-SP-conjugated ChromPure Rabbit IgG whole molecules (Jackson) for 2 h at 4˚C. Captured prey proteins were washed with IPP150 buffer twice, electrophoresed, and detected by using a Molecular Imager FX (Bio-Rad). As a negative control, experiments employing a bait protein excluding Jun sequence were also performed.

# Surface plasmon resonance assay

SPR analyses were performed on BIACORE3000 instrument (Biacore). The cDNA fragments of the screened candidates in the pENTR D-TOPO vector were recombined into pDEST15 vector (Invitrogen) in order to fuse an N-terminal GST-tag as described above. The recombined plasmids were PCR-amplified with the following primer set (forward oligonucleotide: 5’- GGAAGATCTATTTAGGTGACACTATAGAACAACAACAACAACAAACAACAACAAAATGTCCCCTATACTAGGTTATTGG-3’; reverse oligonucleotide: 5’- CATCGATAAGCTTTAATGCGGTAG-3’), and transcribed and translated as above. Each resulting GST-fusion protein was captured on a CM4 sensor chip (Biacore) coupled with goat anti-GST mAb as a ligand. For the coupling reaction, an Amine Coupling Kit and a GST Capture Kit (Biacore) were employed. On the other hand, full-length mouse Jun was sub-cloned into pET15 vector (Novagen) and transformed into BL21. Recombinant Jun protein fused with His-tag was expressed in LB medium with Overnight Express reagent (Novagen), overnight at 37˚C. The Jun protein was collected as inclusion bodies, solubilized in a buffer containing 7 M urea and captured on TALON beads (Takara). The captured Jun was refolded on the TALON column by reducing the urea concentration and eluted with 150 mM imidazole (Shi et al. 2003). This recombinant Jun protein was employed as an analyte. After capture of the ligand proteins on a sensor chip, an excess of recombinant GST (Biacore) was captured on the reference flow-cell and remaining space of the analyzing flow-cell to block non-specific interaction between the analyte and the sensor chip. All interaction analyses were performed in HBS-EP buffer (Biacore).

# Supplementary references

Campbell, N.A. 1996. *Biology,* 4th edition. The Benjamin/Cummings Publishing Company, Inc., New York, USA.

Horisawa, K., Tateyama, S., Ishizaka, M., Matsumura, N., Takashima, H., Miyamoto-Sato, E., Doi, N., and Yanagawa, H. 2004. *In vitro* selection of Jun-associated proteins using mRNA display. *Nucleic Acids Res.* **32**: e169.

Miyamoto-Sato, E., Takashima, H., Fuse, S., Sue, K., Ishizaka, M., Tateyama, S., Horisawa, K., Sawasaki, T., Endo, Y., and Yanagawa, H. 2003. Highly stable and efficient mRNA templates for mRNA-protein fusions and C-terminally labeled proteins. *Nucleic Acids Res.* **31**: e78.

Shi, Y., Jiang, C., Chen, Q. and Tang, H. 2003. One-step on-column affinity refolding purification and functional analysis of recombinant human VDAC1. *Biochem Biophys Res Commun.* **303**: 475-482.
